# Responses of Groundwater Bacterial Communities to Extreme Rainfall in the Western North China Plain Based on 16S rRNA Sequencing

**DOI:** 10.3390/microorganisms14092095

**Published:** 2026-09-19

**Authors:** Yuan Gao, Shengpin Li, Rui An, Yiwei Zhang, Haitao Piao, Tuoya Tai, Qianying Zhu, Qi Su, Yu Fei, Chenglong Han, Wenpeng Li, Kun Liu

**Affiliations:** 1 China Institute of Geo-Environment Monitoring, Beijing 100081, China; 2 School of Water Resources and Environment, China University of Geosciences (Beijing), Beijing 100083, China; 3 Key Laboratory of Mine Ecological Effects and Systematic Restoration, Ministry of Natural Resources, Beijing 100081, China

**Keywords:** extreme rainfall, the North China Plain, bacterial community, groundwater, biogeochemical cycling

## Abstract

Extreme rainfall events significantly affect groundwater systems by altering hydrogeological conditions and accelerating the migration of pollutants, exacerbating the ecological vulnerability of over-exploited areas. However, there is a lack of systematic research on the ecological response mechanisms of microbial communities under extreme rainfall. This study focuses on the over-exploited area in the western North China Plain. Based on 16S rRNA sequencing analysis of 49 groundwater samples collected from the typical alluvial–diluvial plain in the study area impacted by extreme precipitation induced by Typhoon Doksuri, we revealed the effects of rainfall on the taxonomic composition, assembly mechanisms, and environmental drivers of groundwater microbial communities. We found that extreme rainfall influenced the distribution of dominant genera in groundwater and enriched tolerant bacterial communities, further causing a decline in groundwater microbial diversity. Our study confirmed that abundant subcommunities displayed strong microbial collaborations, while rare subcommunities exhibited higher sensitivity and evolved toward opportunistic groups after extreme rainfall. Rainfall influenced the composition of rare taxa, while enhancing the influence of stochastic processes to the assembly of subcommunities. Functionally, confined water favored anaerobic carbon fixation, methanogenesis and dissimilatory sulfate reduction, whereas the Calvin–Benson–Bassham pathway, nitrification, and denitrification were enriched in phreatic water. Additionally, abundant taxa exhibited significantly responses to carbon nutrients in shallow groundwater, whereas rare taxa were governed by mineral ions in deep confined aquifers after rainfall. This study expanded our knowledge of the groundwater microbiome driven by extreme rainfall events and was of great significance to the assessment of ecological stability in the western North China Plain.

## 1. Introduction

The subterranean environment hosts up to 30% of all microbial biomass on Earth. Groundwater microbiomes harbor a remarkable diversity of prokaryotes, microeukaryotes, and viruses. Advances in next-generation sequencing and computational technologies have greatly expanded groundwater microbiome research, resulting in a shift from morphology and physiology to genomics and ecology. This transformation has led to discoveries of new species and an improved understanding of complex interactions among groundwater microorganisms [1,2,3]. Groundwater microbial communities are differentiated into abundant and rare microbial subcommunities. Generally, abundant taxa are thought as most active and important in biogeochemical cycles, whereas rare taxa serve as a seed bank that may become active under favorable conditions for maintaining functional stability and flexibility of ecosystems or habitats [4,5]. The groundwater microbial communities are linked directly to carbon, nitrogen, and sulfur cycling, sustaining the ecosystem health and groundwater quality [6].

The North China Plain (NCP) is a crucial agricultural core region of northern China with a warm and semi-humid continental monsoon climate [7]. Groundwater underpins regional food security and socio-economic development by supporting domestic water supply, agricultural irrigation, and industrial production. The piedmont plain and alluvial fan are located in the western North China Plain. They serve as the primary discharge zones where groundwater, recharged from the piedmont area, first emerges or overflows to the surface or into lower aquifers [8,9,10]. This region is also the most severely over-extracted area in terms of shallow groundwater in the North China Plain [11,12]. Groundwater overexploitation, excessive agricultural fertilization, and leakage of wastewater have altered the regional hydrological processes and hydrogeochemical evolution [13]. Meanwhile, the increasing frequency of extreme rainfall events induces impulse disturbances, reshaping groundwater recharge patterns, hydrochemistry characteristics, and subsurface ecological conditions [14,15,16].

As the reliance on groundwater and climate change continue to increase, analyses based on gene datasets could provide new insights into the responses of microbial diversity and functions to climate changes in groundwater systems [17,18]. Similarly, predictive functional profiling that targets the sequences in samples has been applied to analyze the microbial functions in groundwater, including energy metabolism, organic matter utilization, and microbial interactions [19,20,21,22]. Existing research has confirmed that extreme rainfall introduces exogenous nutrients, organic matter, and surface microorganisms from land to rivers and expand the subsurface genetic library, altering subsurface redox conditions, ionic composition, and contaminant mobilization that drive microbial metabolic and defense functions [23]. The appearance of large-scale depression cones resulting from continuously decreasing groundwater levels during long-term overexploitation in the North China Plain further amplify the bidirectional exchange of microorganisms between groundwater and surface waters [8,24]. Notably, changes in element cycling (e.g., nitrogen cycling, carbon cycling) in aquifers have been observed, and there might be a correlation between the invaded pollutants and altered reactions regulated by groundwater microbiota [25]. Additionally, extreme rainfall regulates the balance between deterministic and stochastic assembly processes and affects spatial patterns of abundant and rare species. Thus, the microbiome composition and function in response to extreme rainfall events in the subsurface aquifers need to be explored for further understanding of groundwater ecosystems in the western North China Plain [26].

**Figure 1:**
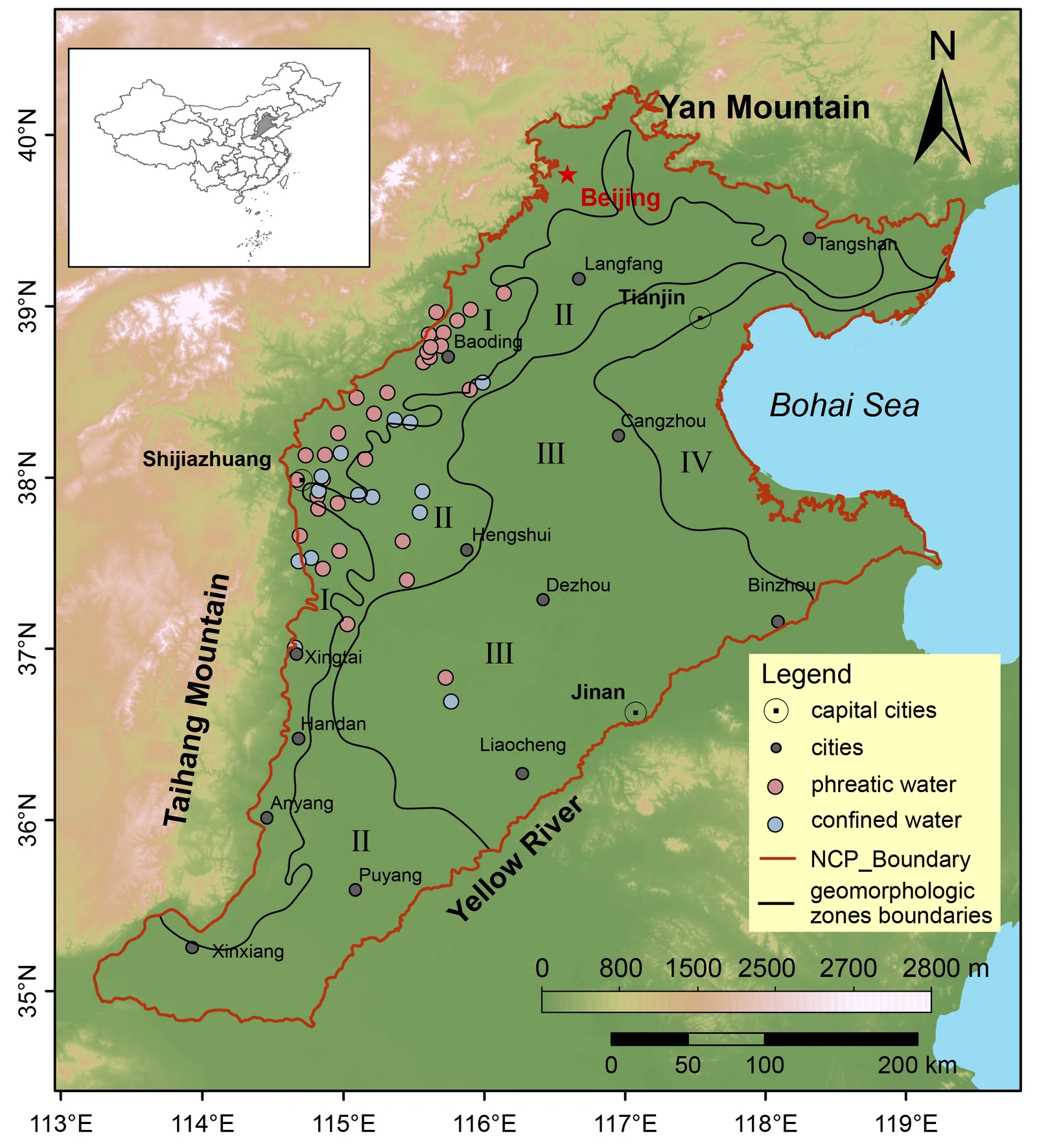
Map of alluvial–diluvial plain areas showing the groundwater sampling sites in the western North China Plain. Boundaries of geomorphologic zones: (I) piedmont plain, (II) alluvial fan, (III) alluvial plain, and (IV) coastal plain.

To fill this gap, we investigated the effects of rainfall on the taxonomic and functional composition, assembly mechanisms, and environmental drivers of groundwater microbial communities based on 16S rRNA sequencing analysis (Figure S1). In this study, we collected 49 groundwater samples from an alluvial–diluvial plain in the western North China Plain after extreme rainfall (Figure 1) and aimed to (a) reveal the effects of rainfall on the bacterial diversity and structures of groundwater; (b) determine the composition and environmental adaptability of rare and abundant bacterial taxa in both phreatic and confined aquifers; (c) elucidate the assembly mechanisms involved in shaping the abundant and rare subcommunities; and (d) identify the microbial functional diversity and the coupled cycling of carbon, nitrogen, and sulfur. The study provides new insights into the adaptive responses of bacterial communities to extreme rainfall in groundwater ecosystems of the North China Plain. This is greatly beneficial for assessing subsurface vulnerability and sustainable potential relevant to various climate disturbances.

## 2. Materials and Methods

### 2.1. Study Area Description and Sampling

The North China Plain (32–40° N, 114–121° E) aquifer extends from the Taihang Mountains on the west to the Bohai Sea on the east [7]. This study area is a typical alluvial–diluvial plain, which has a temperate continental monsoon climate with an annual average precipitation ranging from 500 to 534 mm [8]. Notably, the precipitation is unevenly distributed across seasons, with approximately 75% falling during the summer flood season from July to August [27], thereby leading to the frequent flooding events in the study region. Beginning on 29 July 2023, the NCP experienced extreme heavy precipitation due to the influence of Typhoon Doksuri. Considering typical hydrogeological zones, potential ecologically fragile regions, and frequent flooding events, we collected 49 groundwater samples (33 phreatic and 16 confined groundwater samples) from Shijiazhuang, Baoding, and Xingtai in the western NCP during extreme rainfall. Based on groundwater types identified by the China Geological Survey (https://geocloud.cgs.gov.cn/), these sampling sites included karst water and porous water. All groundwater samples were derived from monitoring wells in the national groundwater monitoring project according to the procedures of national standards (HJ 164-2020) [28].

Before sample collection, sampling wells were flushed with a submersible sampling pump at a controlled discharge below 0.3 L/min. When water yield stabilized and the water became clear, physicochemical properties (i.e., pH, conductivity, and oxidation–reduction potential) of outflowing groundwater were measured with a portable tester for 15 min until three consecutive measurements were consistent (standard deviation < 5%) [29]. Following this purge, 30 L of groundwater were formally extracted, filtered with 0.22 μm polycarbonate membranes (Millipore, Burlington, MA, USA) to enrich microbial cells within 24 h [3,30], and then immediately transported with dry ice to an adjacent laboratory. The filter membranes were kept frozen at −80 °C for DNA extraction.

### 2.2. Groundwater Physicochemical Analysis

At each sampling site, 10 L of the groundwater sample was collected and stored at 4 °C for further physicochemical analysis. For inorganic elements in each sample, an ion chromatograph (Integrion, Thermo Fisher Scientific, Sunnyvale, CA, USA) was used, referring to the Chinese Ministry of Ecology and Environment, who issued the official water quality inspection standard document HJ 84-2016 [31] “Determination of Inorganic Anions in Water Quality by Ion Chromatography Method”, to detect and analyze four anions: fluoride (F^−^), chloride (Cl^−^), sulfate (SO_4_^2−^), and nitrate (NO_3_^−^). Using the inductively coupled plasma optical emission spectrometry (ICP OES) (OPTIMA 8300, PerkinElmer Scientific, Singapore) and referring to HJ 776-2015 [32] “Determination of 32 Elements in Water Quality by Inductively Coupled Plasma Optical Emission Spectrometry”, potassium (K), calcium (Ca), sodium (Na), and magnesium (Mg) were detected and analyzed. Iron (Fe), manganese (Mn), copper (Cu), zinc (Zn), and aluminum (Al) were detected and analyzed using inductively coupled plasma-mass spectrometry (NexION 350D, PerkinElmer Scientific, Singapore), following HJ 700-2014 [33] “Determination of 65 Elements in Water Quality by Inductively Coupled Plasma Mass Spectrometry”. Arsenic (As) and selenium (Se) in groundwater were determined by atomic fluorescence spectrometry (AFS-9530, Haiguang, Beijing, China) and referring to HJ 694-2014 [34] “Determination of 5 Elements in Water Quality by Atomic Fluorescence Spectrometry”. Using a UV 1800 spectrophotometer (Shimadzu, Kyoto, Japan) and referring to DZ/T 0064.60-2021 [35] “Methods for Analysis of Groundwater Quality Part 60: Determination of Nitrite by Spectrophotometer”, nitrite (NO_2_^−^) was detected and analyzed. A potentiometric titrator (Shimadzu, Kyoto, Japan) was used to detect and analyze bicarbonate (HCO_3_^−^) in accordance with DZ/T 0064.49-2021 [36] “Methods for Analysis of Groundwater Quality Part 49: Determination of Carbonate, Bicarbonate, and Hydroxide Ions by Titration”. Using a UV 1800 spectrophotometer (Shimadzu, Kyoto, Japan) and referring to HJ 535-2009 [37] “Determination of Ammonia nitrogen in Water Quality by Nessler’s Reagent Spectrophotometry” and DZ/T 0064.17-2021 [38] “Methods for Analysis of Groundwater Quality Part 17: Determination of Total Chromium and Chromium (VI) Content by 1,5 Diphenylcarbahydrazide Spectrophotometry”, ammonia nitrogen (NH_4_^+^-N) and chromium (Cr) were detected and analyzed. Referring to DZ/T 0064.68-2021 [39] “Methods for Analysis of Groundwater Quality Part 68: Determination of Oxygen Consumption by Acidic Permanganate Titrimetric Method”, oxygen consumption (COD_Mn_) was detected and analyzed. Total hardness was detected and analyzed with reference to DZ/T 0064.15-2021 [40] “Methods for Analysis of Groundwater Quality Part 15: Determination of Total Hardness by EDTA Titrimetric Method”. The Total Dissolved Solids (TDS) was analyzed using the gravimetric method in accordance with DZ/T 0064.9-2021 [41] “Methods for Analysis of Groundwater Quality Part 9: Determination of Total Dissolved Solids by Gravimetric Method”. Using a pH meter (S220, Mettler toledo, Greifensee, Switzerland) and referring to DZ/T 0064.5-2021 [42] “Methods for Analysis of Groundwater Quality Part 5: Determination of pH by Glass-electrodes Method”, the pH was detected.

For each sampling site, the longitude and latitude were recorded using a handheld GPS (Magellan, Thermo Fisher Scientific, Hillsboro, OR, USA) during sample collection. The standards referenced were presented in Table S1. Details of each method were described in the Supplemental Methods (Supplementary Information).

### 2.3. DNA Extraction, Target-Gene Amplification, and Sequencing

The FastDNA^®^ SPIN Kit for Soil (MP Biomedicals, Solon, OH, USA) was used to isolate genomic DNA from each sample as per the manufacturer’s protocol. The DNA quality was evaluated using a NanoDrop ND-2000 instrument (Thermo Fisher Scientific, Wilmington, DE, USA), and high-quality DNA (DNA amount > 1 μg and concentration > 10 ng/μL) was retained for subsequent experiments [30]. The barcoded primer pairs F (5′-GTGCCAGCMGCCGCGGTAA-3′) and R (5′-GGACTACHVGGGTWTCTAAT-3′) were used to amplify the bacterial 16S rRNA genes [43]. The PCR reaction was conducted at 95 °C for 3 min; 29 cycles of 95 °C for 30 s, 55 °C for 30 s, and 72 °C for 45 s; then 72 °C for 5 min. Based on the concentration to generate amplicon libraries, PCR products were sequenced on an Illumina MiSeq PE300 platform (Illumina Inc., San Diego, CA, USA, Table S2) at Meige Gene Technology Co., Ltd. (Guangzhou, Guangdong Province, China).

### 2.4. Bioinformatics Analyses

Raw sequencing reads were quality-filtered using the Fastp v0.14.1 (-q 15 -u 40) [44]. High-quality data were assembled with USEARCH v10.0.240 [45]. Operational taxonomic units (OTUs) were clustered with 97% similarity cutoff using UPARSE (version 7.1) [46], and chimeras were detected and removed using the UCHIME algorithm. The taxonomy of each representative sequence was assigned using RDP Classifier v2.26 based on a 16S rRNA gene database (http://www.arb-silva.de/) with a confidence threshold of 0.7 [47,48]. OTUs were identified at the level of phylum, family, order, class, and genus. To investigate the community dynamics of abundant (AT) and rare taxa (RT) as well as their responses to extreme rainfall events, we defined the relative abundance thresholds of 1% and 0.01% for abundant and rare taxa, respectively [49]. Bacterial communities were classified into six taxonomic groups: (i) always abundant taxa (AAT), (ii) conditionally abundant taxa (CAT), (iii) conditionally abundant or rare taxa (CRAT), (iv) moderate taxa (MT), (v) conditionally rare taxa (CRT), and (vi) rare taxa (RT) as previous studies reported [50,51]. Abundant taxa were defined as the assemblage of OTUs (i.e., AAT, CAT, and CRAT) whose relative abundance exceeded 1% in at least one sample [51]. In this study, predictive functional profiling was performed using Phylogenetic Investigation of Communities by Reconstruction of Unobserved States (PICRUSt) [52,53]. Functional profiles were classified into different functional categories based on the Kyoto Encyclopedia of Genes and Genomes (KEGG) database [54].

### 2.5. Statistical Analyses

Bacterial richness and alpha-diversity indices, including, Chao1, Shannon, and Abundance-based Coverage Estimator (ACE), were calculated for each sample. The Kruskal–Wallis test was used to compare the significance of group differences. Spearman’s rank correlations were calculated to further explore the interaction patterns of abundant and rare taxa based on statistical significance (correlation coefficient > 0.7 and FDR-adjusted *p* < 0.01) [55]. Moreover, networks were visualized, and corresponding node-level topological properties (i.e., weighted degree and closeness centrality) were calculated using Gephi (v0.9.2) and R (v4.1.0) packages [56]. Pearson and Spearman correlation analyses were performed using SPSS software (v26) (IBM Corporation, Armonk, NY, USA), and visualization was plotted using R (v4.1.0) with the *ggplot2* package (v3.3.5).

Based on Bray–Curtis distance matrices of bacterial communities among samples, constrained ordination was performed using non-metric multidimensional scaling (NMDS). Analysis of similarity (ANOSIM) was calculated to test the significance of differences in community diversity and structures among specific groups using R with the *vegan* package (v2.5.6). Linear discriminant analysis Effect Size (LEfSe) [57] was used with Wilcoxon and Kruskal–Wallis tests to discover biomarkers and characterize taxa differences for different groups.

The Sloan neutral community model was used to estimate the potential role of neutral processes in shaping the bacterial community structure [58]. We estimated the relative importance of deterministic and stochastic processes in R (v4.1.0) using the *NST* package (v3.1.10) based on the normalized stochasticity ratio (NST), whose values differentiate assemblies in bacterial communities dominated by deterministic processes (<50%) and stochastic processes (>50%) [59,60].

## 3. Results

### 3.1. Rainfall-Induced Changes in the Physicochemical Parameters and Hydrochemical Types

**Figure 2:**
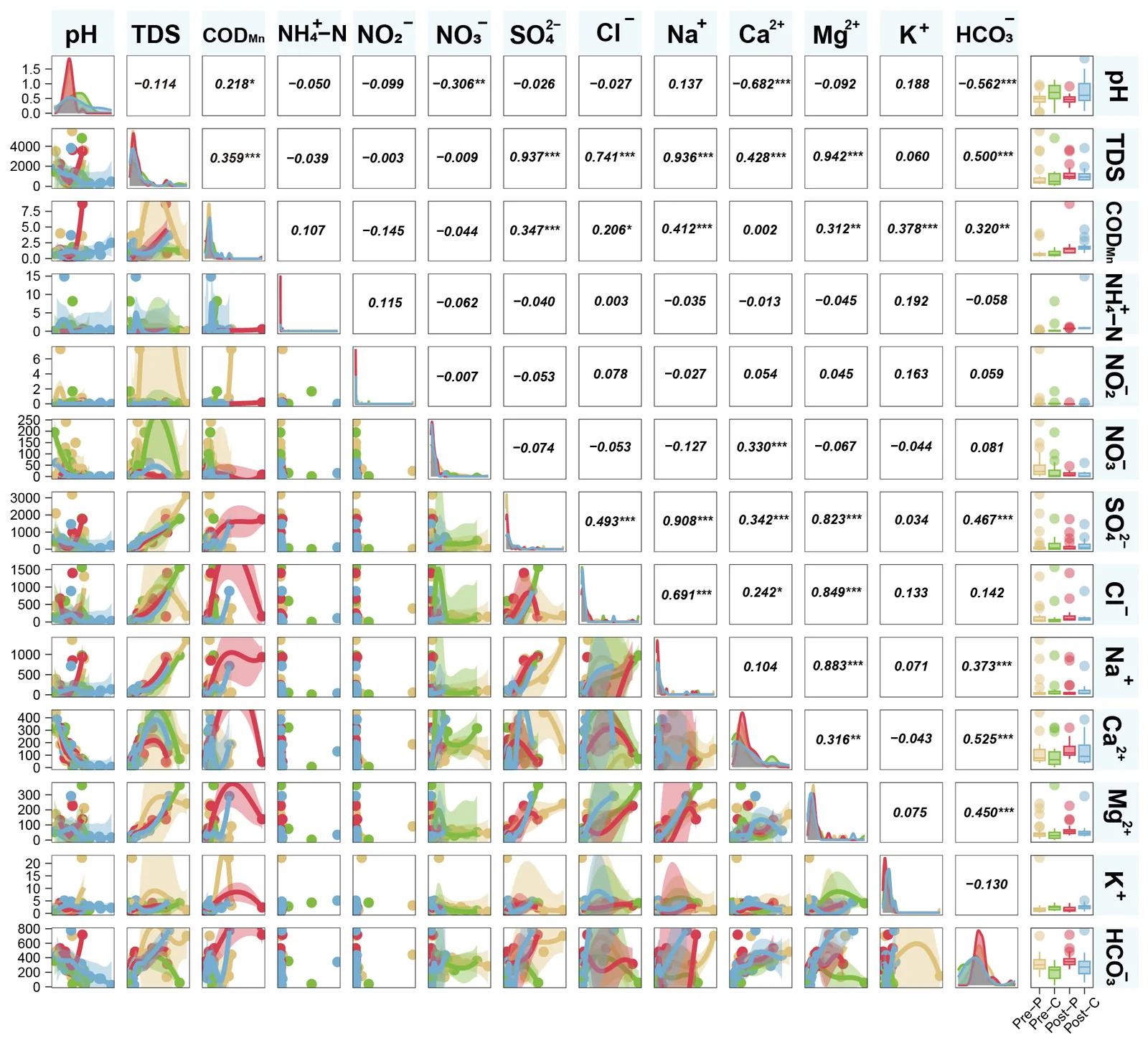
Correlations between the environmental variables in phreatic and confined water before and after extreme rainfall. The lower corner on the left shows the data distribution of environmental variables. The upper-right corner indicates the correlation coefficient and *p*-values. The asterisk (*) represented *p*-values (*, *p*-value ≤ 0.05; **, *p*-value ≤ 0.01; ***, *p*-value ≤ 0.001). Pre_P, the samples in phreatic water before extreme rainfall; Pre_C, the samples in confined water before extreme rainfall; Post_P, the samples in phreatic water after extreme rainfall; Post_C, the samples in confined water after extreme rainfall.

Variations in the groundwater physicochemical parameters after extreme rainfall are presented in Figure 2. Rainfall increased the TDS (803.79 mg/L), NH_4_^+^-N (0.37 mg/L), and COD_Mn_ (0.85 mg/L) compared to those before rainfall, whereas it decreased the NO_3_^−^-N (10.22 mg/L). TDS was strongly and positively correlated with the content of COD_Mn_, Ca^2+^, Na^+^, Mg^2+^, Cl^−^, SO_4_^2−^, and HCO_3_^−^. The groundwater samples were weakly alkaline with an average pH of 7.67, in line with the higher HCO_3_^−^ accumulation in the study area after rainfall, which was mainly related to chemical erosion of carbonate minerals. Fe (0.22 mg/L) and Mn (0.05 mg/L) were the dominant trace metals in groundwater. Ca^2+^ (108.82 mg/L), Na^+^ (96.94 mg/L), and Mg^2+^ (48.40 mg/L) were identified as major cations while HCO_3_^−^ (308.63 mg/L) and SO_4_^2−^ (202.50 mg/L) were dominant anions.

**Figure 3:**
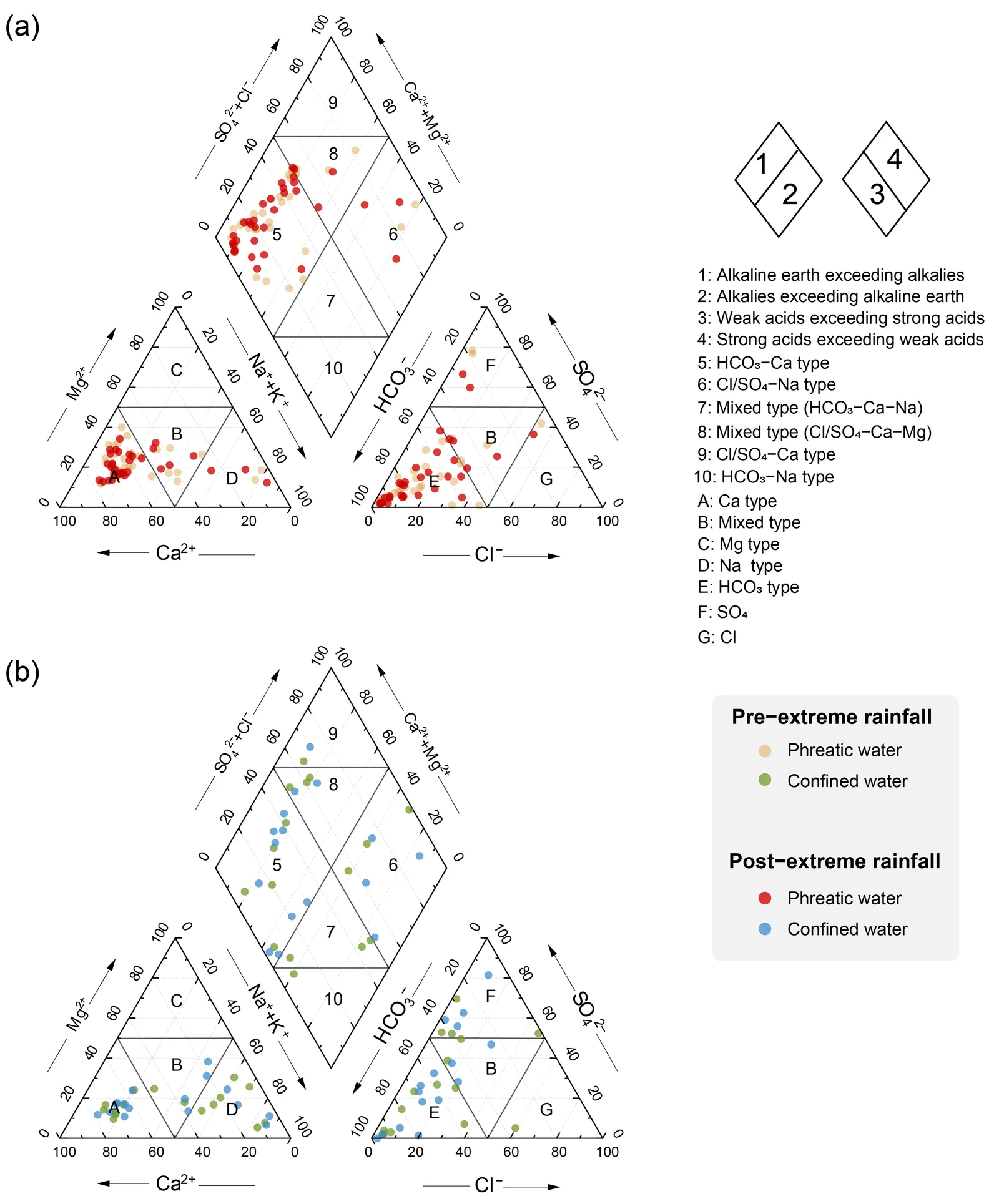
Piper diagrams showing the hydrochemical types of groundwater. (**a**) Phreatic water. (**b**) Confined water.

A Piper diagram was generated to identify the hydrochemical types of groundwater in the western North China Plain driven by extreme rainfall. The hydrochemical types of groundwater samples in phreatic water included HCO_3_-Ca, mixed Cl⋅SO_4_-Ca⋅Mg, and Cl⋅SO_4_-Na (Figure 3a). Most of the groundwater samples in phreatic water plotted in the HCO_3_-Ca zone of the Piper diagram before and after extreme rainfall. Before rainfall, Piper analysis further showed that the hydrochemical types of the groundwater samples in confined water were assigned to HCO_3_-Ca, mixed Cl⋅SO_4_-Ca⋅Mg, mixed HCO_3_-Ca⋅Mg, Cl⋅SO_4_-Na, and Cl⋅SO_4_-Ca types (Figure 3b), while the primary hydrochemical types gradually evolved to HCO_3_-Ca after rainfall.

### 3.2. Diversity, Structure, and Potential Function of the Bacterial Community

**Figure 4:**
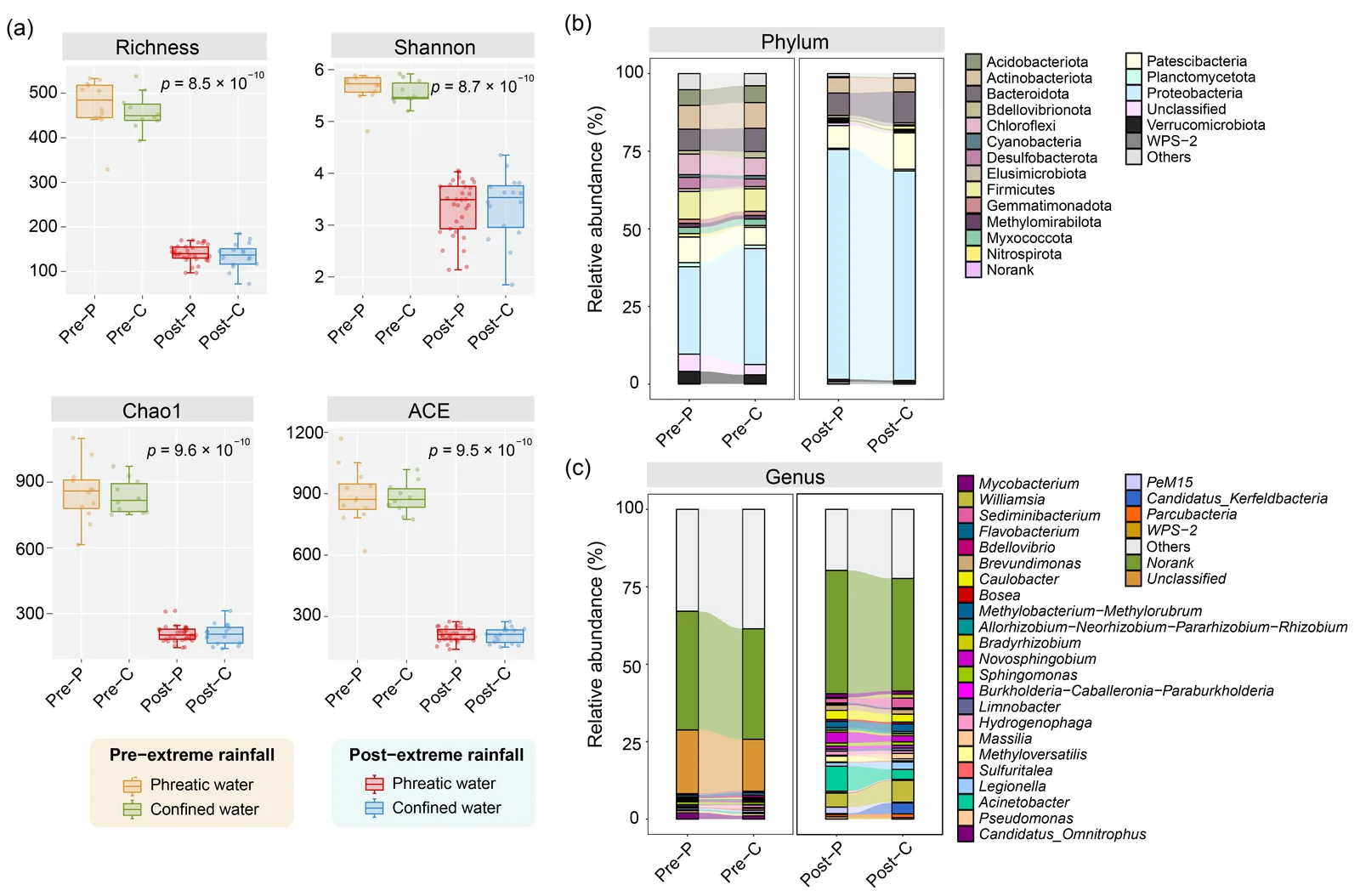
Diversity and composition of bacterial communities in phreatic and confined water before and after extreme rainfall. (**a**) Boxplots showing richness, Shannon index, Chao1, and ACE of bacteria in phreatic and confined water before and after extreme rainfall. Boxplots are represented with center line: median; box limits: upper and lower quartiles; whiskers: 1.5 × interquartile range. The composition of bacterial communities at the phylum (**b**) and genus (**c**) level.

Illumina high-throughput sequencing yielded a total of 6,490,931 raw reads from 49 groundwater samples in the western North China Plain. After quality filtration and elimination of chimeras, the rarefaction curves of each sample indicated that the existing sequencing depth sufficiently captured the majority of bacterial taxa (Figure S2). The bacterial alpha diversity in phreatic and confined water, including Shannon diversity, Chao1, ACE, and richness, decreased significantly after extreme rainfall (Figure 4a).

Among the 71 identified phyla, Proteobacteria was the most dominant phylum, accounting for 28.11~37.30% and 67.47~74.03% of the total OTUs before and after rainfall, respectively, followed by Patescibacteria, Actinobacteriota, Firmicutes, and Bacteroidota (Figure 4b, Table S3). NMDS and ANOSIM analyses illustrated that extreme rainfall induced significant discrepancy in bacterial composition between phreatic and confined water from the western North China Plain (Figure S3, Anosim test, R = 0.673, *p* < 0.001). At the genus level, the genera with high relative abundances before extreme rainfall were *Candidatus_Omnitrophus*, *Sphingomonas*, *Pseudomonas*, *Flavobacterium*, and *Hydrogenophaga*, whereas *Acinetobacter*, *Novosphingobium*, *Caulobacter*, *Methylobacterium*, *Methyloversatilis*, *Brevundimonas*, *Sediminibacterium*, *Candidatus_Kerfeldbacteria*, *Legionella*, and *Massilia* were relatively abundant after rainfall (Figure 4c, Table S4). This indicates that extreme rainfall can influence the distribution of dominant taxa in groundwater.

**Figure 5:**
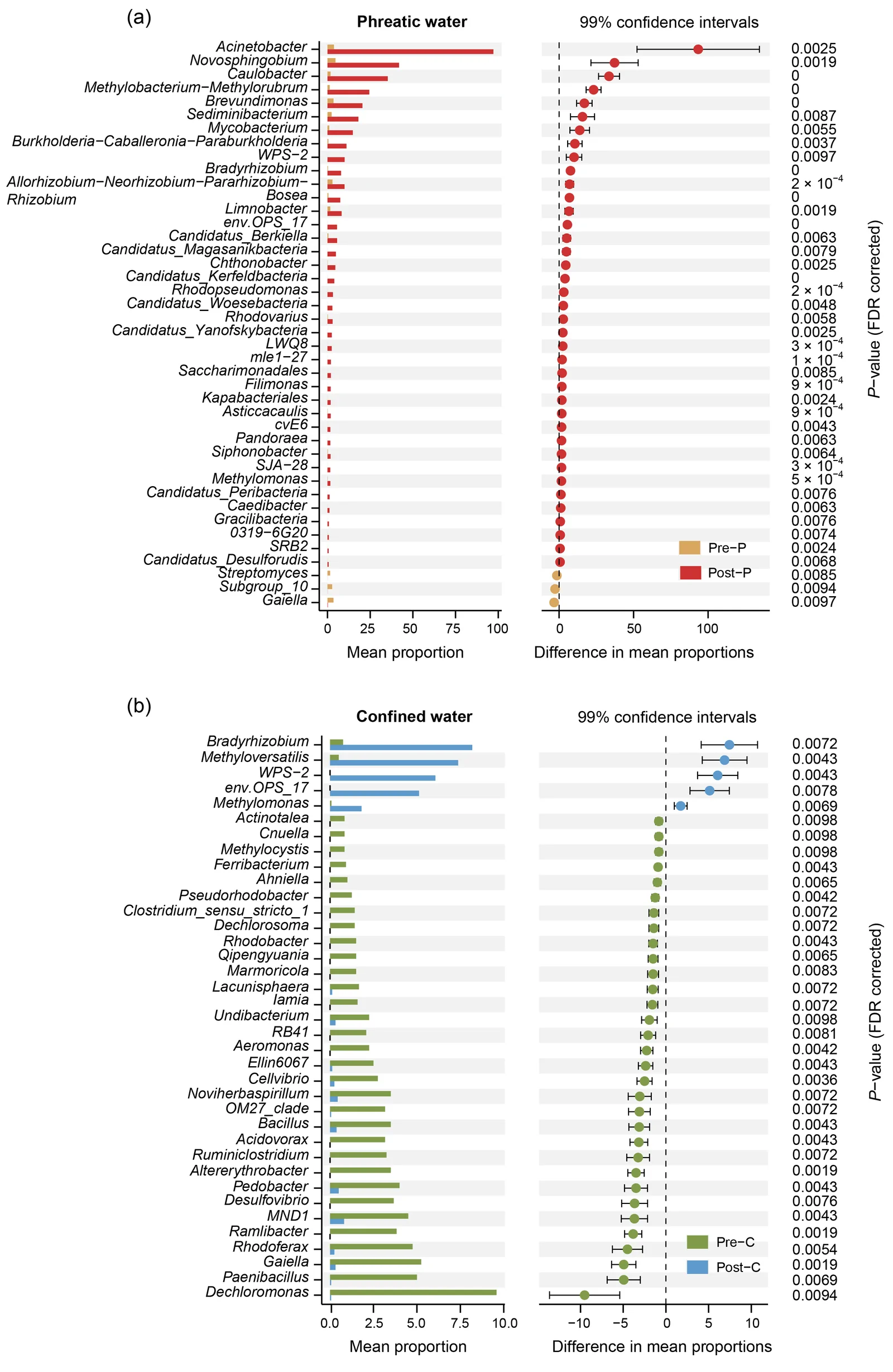
Comparison of groundwater bacterial communities in the western North China Plain before and after extreme rainfall. (**a**) Phreatic water; (**b**) Confined water.

The disturbance caused by extreme rainfall on some bacteria in different aquifers showed distinct differences. The relative abundance of *Acinetobacter*, *Novosphingobium*, *Caulobacter*, *Methylobacterium*, *Brevundimonas*, *Sediminibacterium*, *Mycobacterium*, and other genera increased significantly, while *Streptomyces* and *Gaiella* were slightly affected in phreatic water (Figure 5a). Within the confined water, *Bradyrhizobium*, *Methyloversatilis* and *Methylomonas* were distinctly enriched, and the community structures of *Dechloromonas*, *Paenibacillus*, *Rhodoferax*, *Ramlibacter*, *Desulfovibrio*, and other taxa remained relatively stable (Figure 5b). It revealed that extreme rainfall results in the enrichment of tolerant bacterial communities, further causing a decline in microbial diversity. Furthermore, owing to stronger connectivity with the surface, bacterial communities in phreatic aquifers are more susceptible to disturbances induced by rainfall.

**Figure 6:**
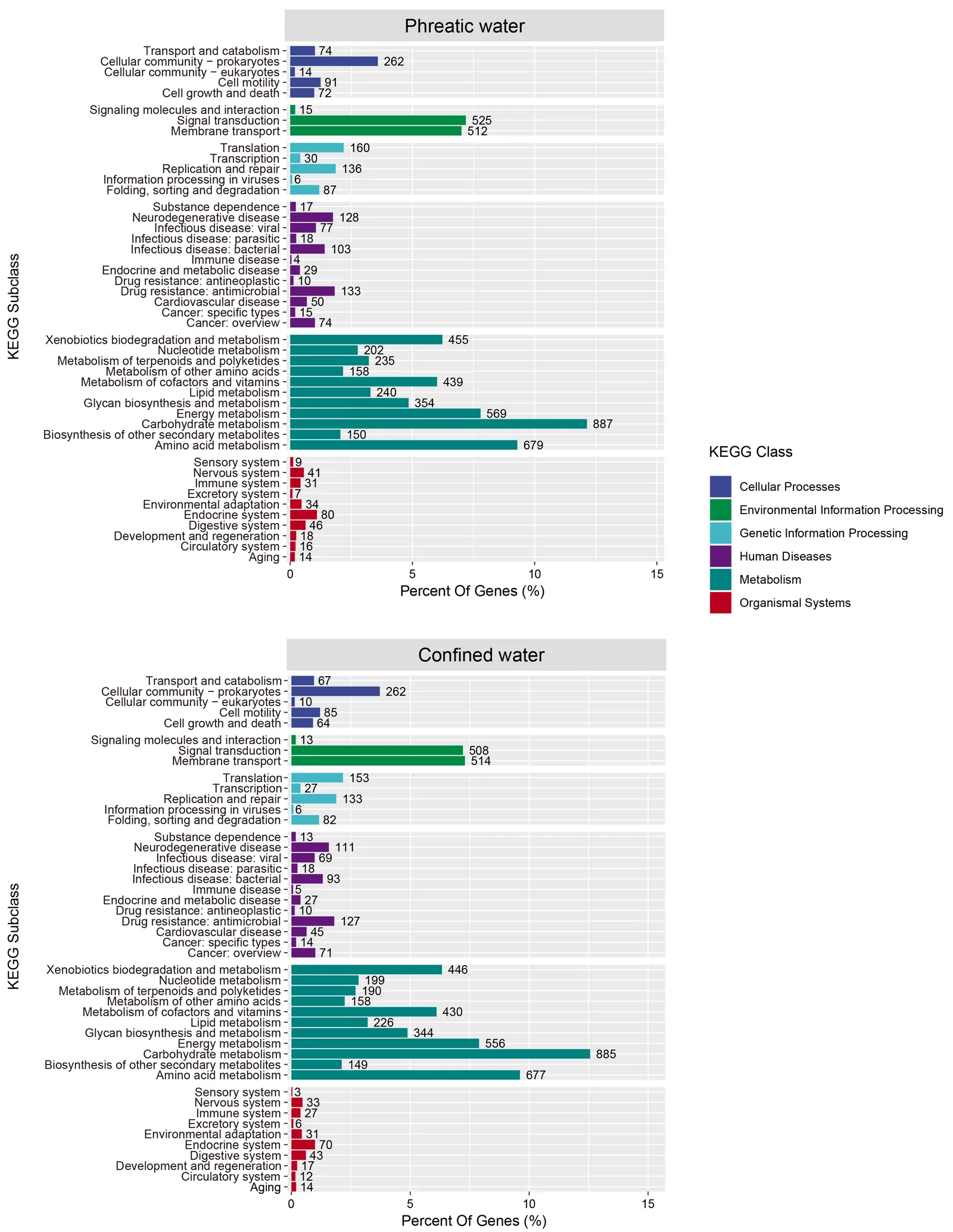
Bacterial functional profiles in phreatic and confined aquifers of the western North China Plain.

Moreover, identified functional genes were associated with metabolism, cellular processes, genetic information processing, environmental information processing, human disease, and organismal systems (Figure 6). In both phreatic and confined aquifers, functional genes were predominantly involved in metabolism (carbohydrate metabolism and energy metabolism) and environmental adaptation (membrane transport and signal transduction), alongside xenobiotic biodegradation and metabolism, suggesting that bacteria inhabiting both phreatic and confined aquifers adopted similar survival strategies in such oligotrophic and anoxic subterranean environments.

### 3.3. Abundant and Rare Subcommunities and Their Responses to Extreme Rainfall Events

**Figure 7:**
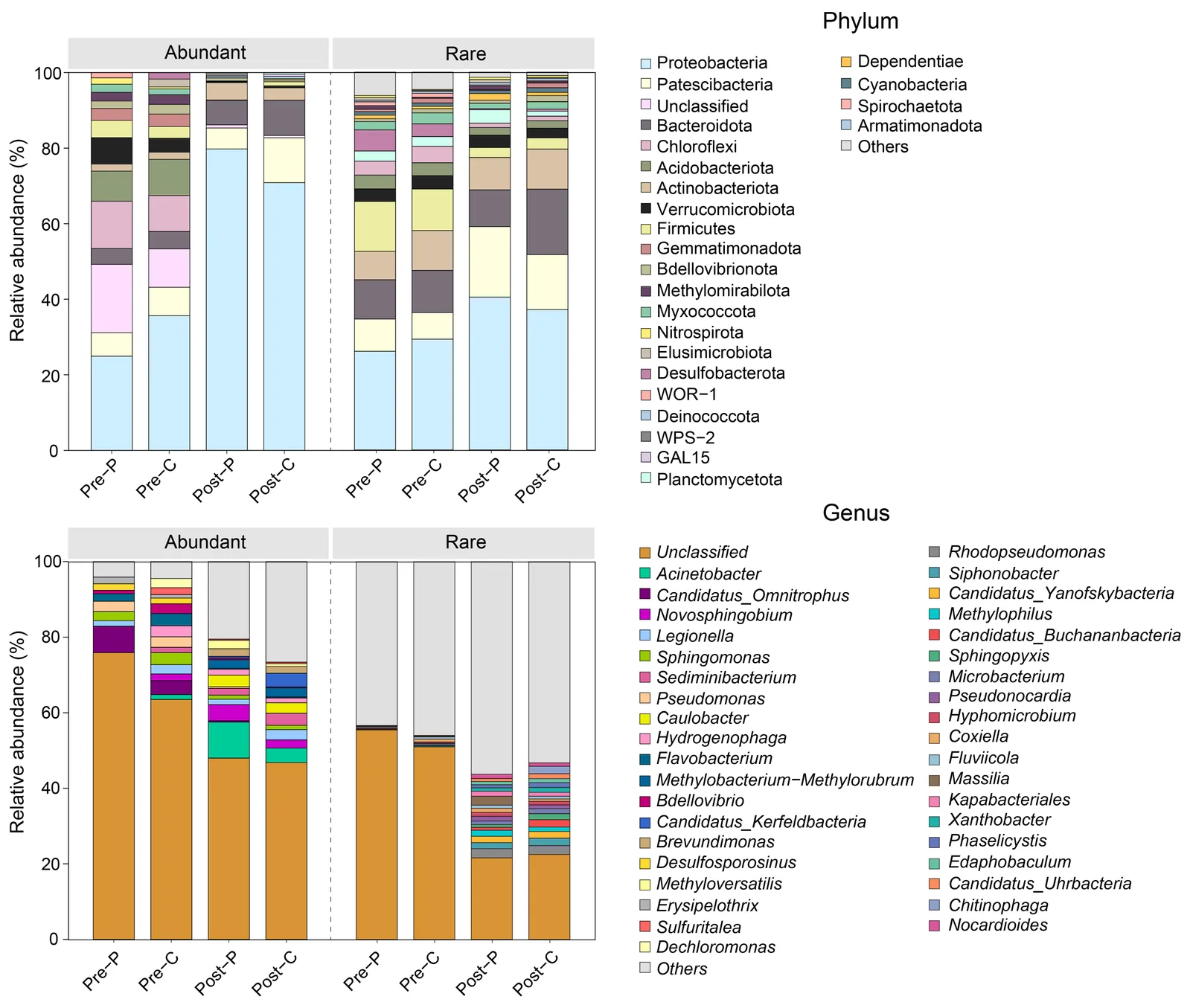
The composition of abundant and rare subcommunities in phreatic and confined water before and after extreme rainfall.

Before the extreme rainfall, abundant taxa (AT, only accounting for 2.18% and 2.76% of all OTUs, respectively) were identified in the phreatic and confined aquifers. Rare taxa (RT) accounted for 92.12% and 89.70% of total OTUs in the phreatic and confined aquifers, respectively. While AT (8.46% and 10.91% of the OTUs, separately) and RT (90.15% and 86.95% of the OTUs, separately) were identified in the phreatic and confined aquifers after extreme rainfall. As shown in Figure S4, the alpha diversity of AT was significantly increased (*p* < 0.001) after rainfall, suggesting that abundant taxa were the main driver of community differences in groundwater. Conversely, rare taxa exhibited much lower diversity than before rainfall. Proteobacteria was the most abundant phylum in both abundant and rare subcommunities, though the relative abundance of AT and RT after rainfall were higher than those before rainfall (Table S5). Most members of Chloroflexi and Acidobacteriota were identified as AT (22.09%) rather than RT (8.03%), while Patescibacteria, Bacteroidota, and Firmicutes flourished more in RT (49.26%) than in AT (25.83%) (Figure 7). After extreme rainfall, *Acinetobacter*, *Brevundimonas*, *Legionella*, and *Pseudonocardia* all belonging to pathogenic bacteria, preferred the phreatic water. Compared to AT, RT was more sensitive to extreme rainfall. *Rhodopseudomonas*, *Massilia*, *Siphonobacter*, and *Methylophilus* were relatively abundant in rare subcommunities (Table S6).

**Figure 8:**
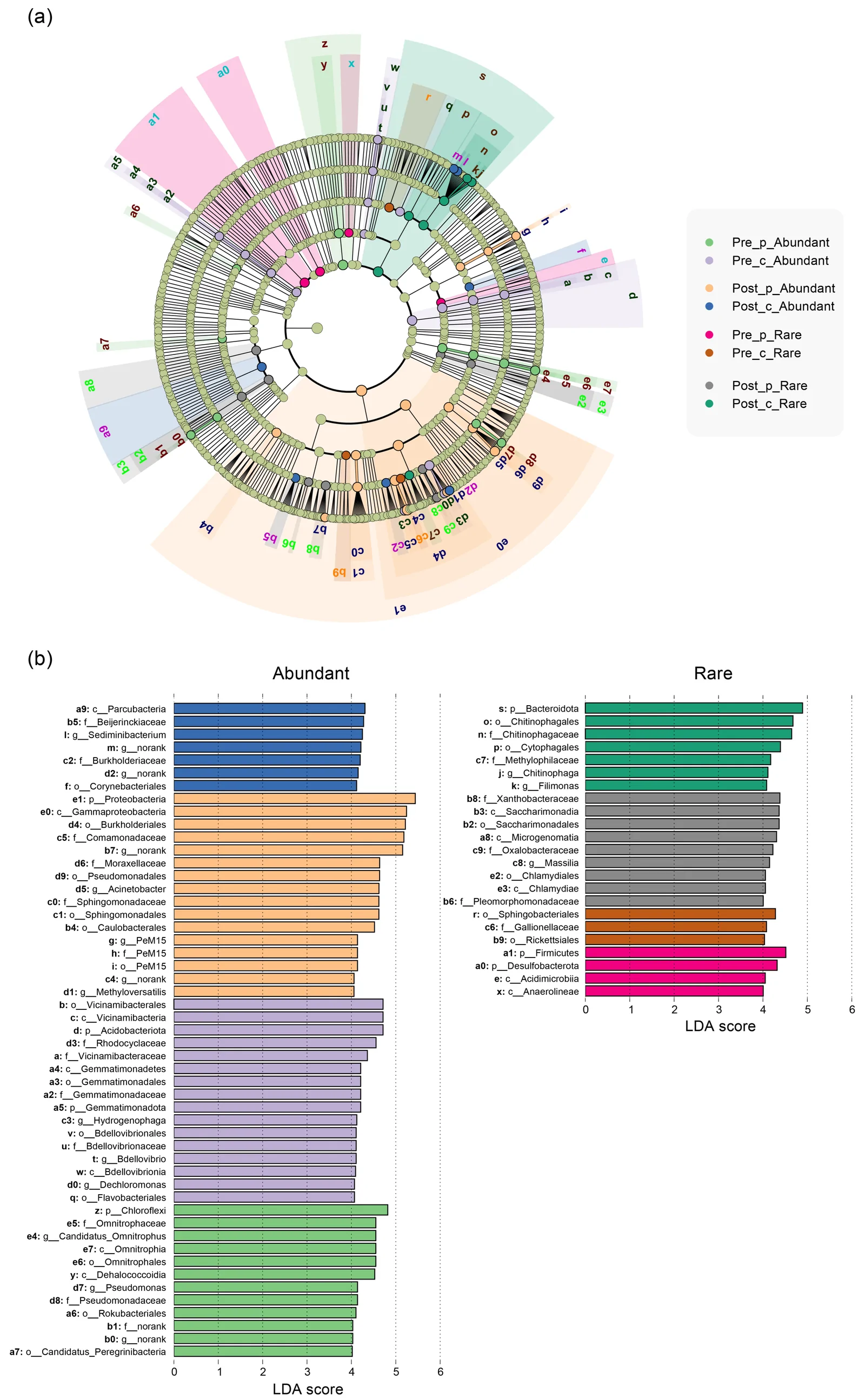
The compositional differentiation of abundant and rare subcommunities in phreatic and confined water before and after extreme rainfall. (**a**) Linear discriminant analysis effect size (LEfSe) cladogram depicting the taxonomic differences of abundant and rare taxa. Differentially abundant taxa (biomarkers) are colored according to their most abundant groups. All detected taxa are assigned to phylum, class, order, family, and genus (outermost). (**b**) Taxa meeting an LDA significant threshold of >4.0 are selected as most likely to explain differences in bacterial communities between different groups.

LEfSe analysis was then used to investigate the compositional differentiation and dominant clades (biomarkers) of abundant and rare subcommunities in phreatic and confined water before and after extreme rainfall (Figure 8). Oligotrophic groups such as *Vicinamibacterales*, *Rhodocyclaceae*, *Peregrinibacteria*, and Omnitrophia played a predominant role in abundant taxa before rainfall, whereas *Gammaproteobacteria*, *Comamonadaceae*, *Moraxellaceae*, *Beijerinckiaceae*, and *Burkholderiaceae* were abundant in abundant taxa after rainfall. This indicated that a large number of heterotrophic groups became dominant in response to increased input of organics and nutrients by rainfall. Meanwhile, *Sphingobacteriales*, *Rickettsiales*, *Acidimicrobiia*, and *Anaerolineae* were prevalent among rare taxa before rainfall, while *Bacteroidota*, *Xanthobacteraceae*, *Saccharimonadales*, *Massilia*, and *Chlamydiae* showed a prevalence in rare taxa after rainfall, indicating that the rare subcommunity was evolving towards opportunistic groups.

Furthermore, we explored how environmental variables influence the community by unraveling the responses of abundant and rare taxa to environmental changes after rainfall (Figure S5). As a result, abundant taxa exhibited significant responses to environmental variation, which were strongly correlated with HCO_3_^−^ and COD_Mn_ in phreatic water, while species belonging to abundant taxa were little affected by environmental factors in confined water. Meanwhile, rare taxa were little affected by environmental factors in phreatic water, while rare taxa exhibited responses to environmental variation, which were significantly correlated with pH, total hardness, TDS, Ca^2+^, Mg^2+^, K^+^, SO_4_^2−^, and F^−^ in confined water. Conversely, bacterial communities that did not experience extreme rainfall were influenced by geographic factors (i.e., longitude, latitude, and depth).

### 3.4. Co-Occurrence Networks and Assembly Processes of Bacterial Community

**Figure 9:**
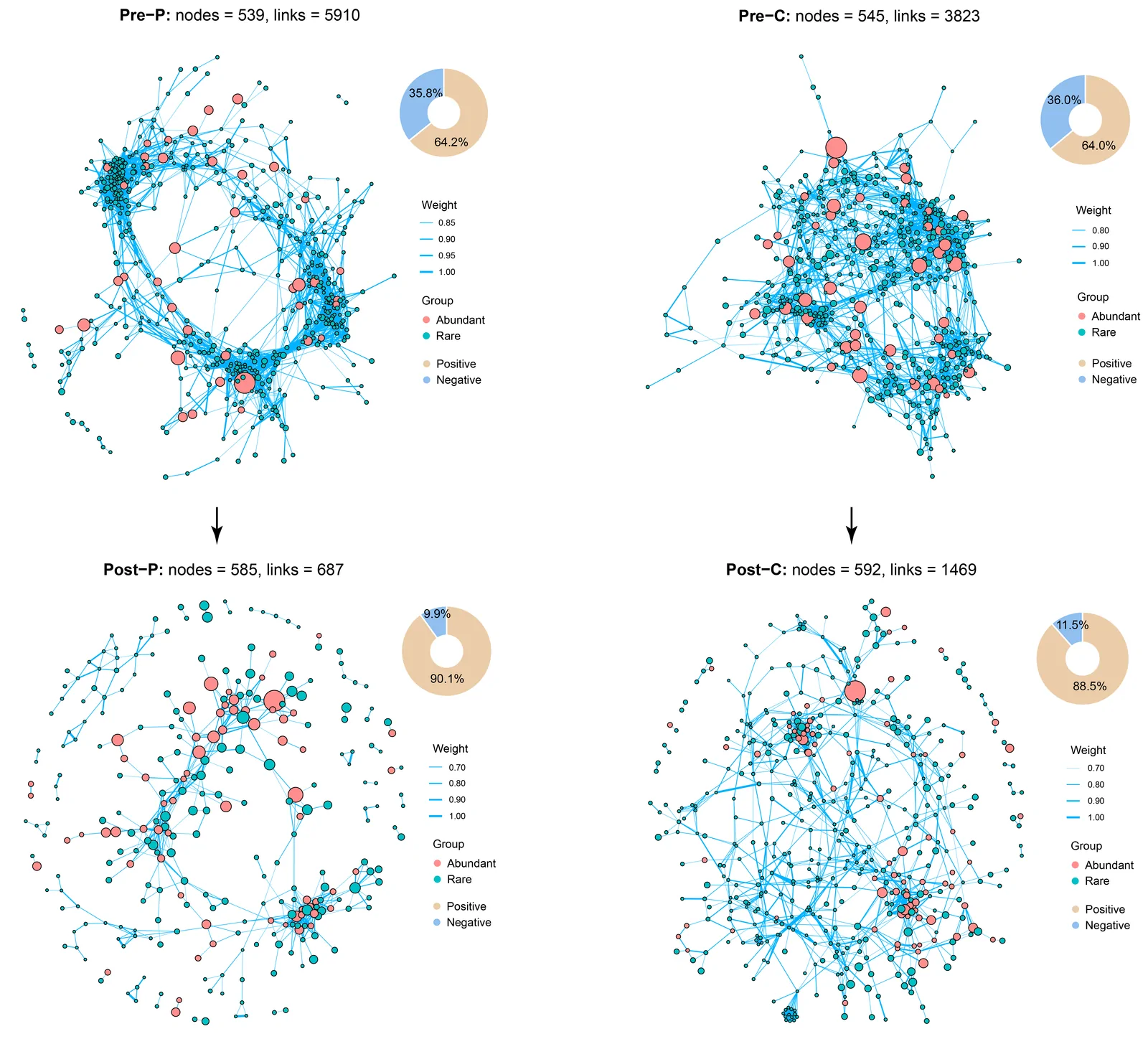
The co-occurrence network of abundant and rare taxa in phreatic and confined water before and after extreme rainfall. The sizes of nodes correspond to the degree of connectivity.

The bacterial co-occurrence networks were constructed based on Spearman’s correlations (|r| > 0.7 and FDR-adjusted *p* < 0.01, Figure 9) to reveal the ecological roles and interrelationships of abundant and rare taxa in phreatic and confined water before and after extreme rainfall. Positive and negative interactions within co-occurrence networks have previously been found to reflect potential mutualistic and antagonistic relationships among microbes [61]. Before extreme rainfall, co-occurrence networks were established by 539 nodes and 5910 links (correlations) for the phreatic water (64.2% positive links) and 545 nodes and 3823 links (64.0% positive links) for the confined water. Rare taxa served as nonnegligible components in both aquifers, forming complex interaction networks where competition and cooperation coexist among species (Table S7).

Notably, co-occurrence networks were established by 585 nodes and 687 links for the phreatic water and 592 nodes and 1469 links for the confined water after rainfall. Network analysis demonstrated a relatively high degree of modularity in aquifers (Table S7). Both aquifers displayed strong microbial collaborations among abundant taxa, as observed by 90.1% and 88.5% positive links. Abundant taxa characterized by a higher weighted degree, whereas rare taxa exhibited a higher closeness centrality (Figure S6), which indicated that AT and RT play important roles in maintaining bacterial community stability in groundwater. Network nodes are characterized by within-module connectivity (*Zi*) and among-module connectivity (*Pi*). The network hub (*Zi* > 2.5, *Pi* > 0.62), module hub (*Zi* > 2.5), and connector (*Pi* > 0.62) could be identified as the keystone taxa of the network. The keystone taxa (16 module hubs and 7 connectors) were composed of Proteobacteria (6), Firmicutes (5), Actinobacteriota (3), Desulfobacterota (2), Myxococcota (2) Bacteroidota (1), Chloroflexi (1), Cyanobacteria (1), Dadabacteria (1), and Planctomycetota (1).

**Figure 10:**
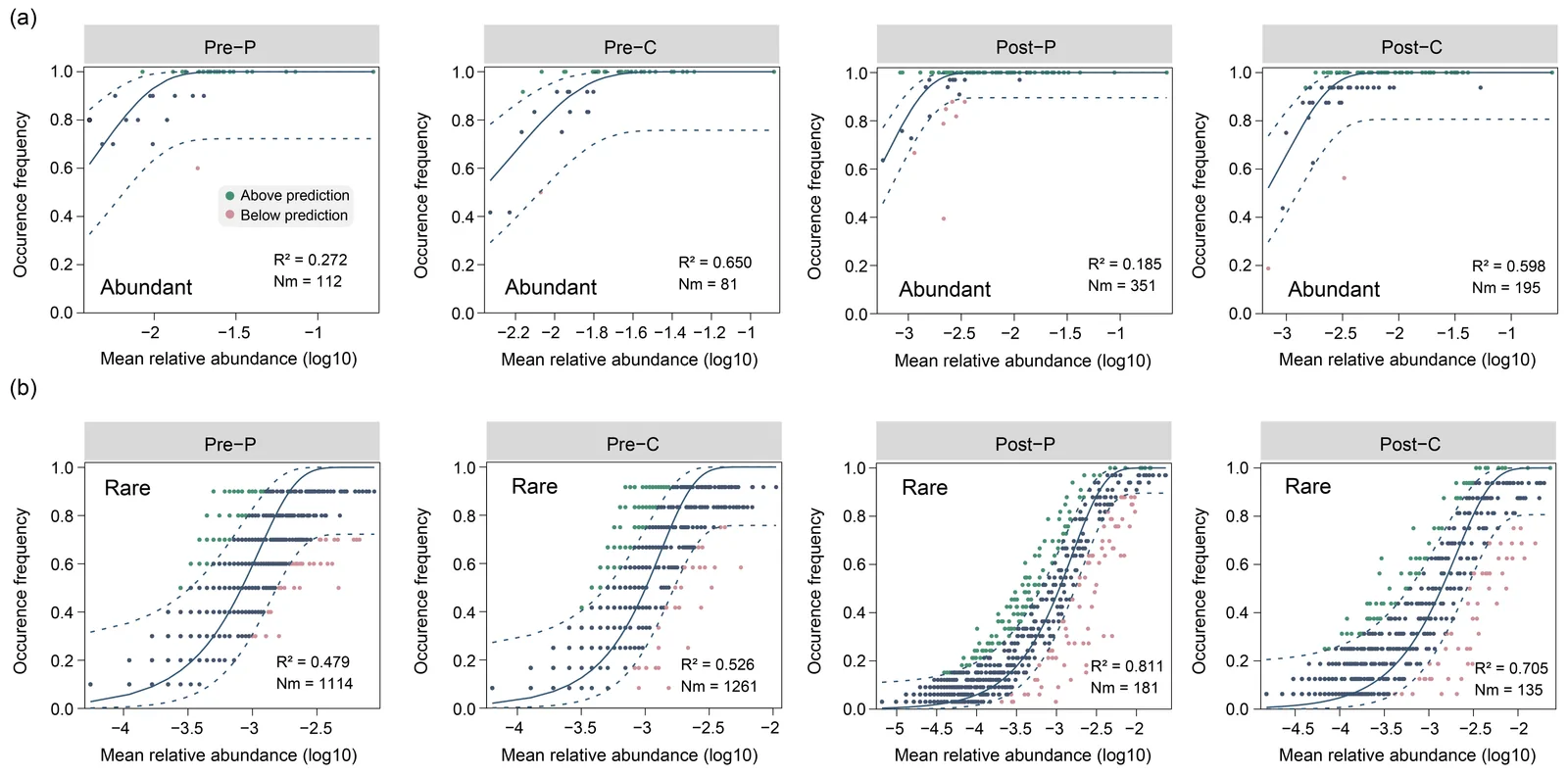
Neutral models evaluating the role of neutral processes in the community assembly of bacteria in phreatic and confined water before and after extreme rainfall. (**a**) Abundant taxa. (**b**) Rare taxa. Blue dashed lines represent 95% confidence intervals of the neutral model prediction, R^2^ is the model goodness of fit. Green and red dots indicate abundant and rare taxa that occur more (‘above’) and less (‘below’) frequently than given by the neutral model (blue dots, ‘neutral’).

To investigate the underlying mechanisms of coexistence and spatial distribution of bacterial communities in phreatic and confined water before and after extreme rainfall, we assessed the contributions of deterministic and stochastic processes in the community assembly of bacteria by the ecological models. The neutral interpretation of the whole bacterial community in phreatic (R^2^ = 0.635) and confined water (R^2^ = 0.661) before the extreme rainfall was lower than those after the extreme rainfall (phreatic: R^2^ = 0.873; confined: R^2^ = 0.812; Figure S7). This indicated that groundwater bacteria suffered a stronger dispersal limitation before rainfall than after rainfall. Based on values of normalized stochasticity ratios (NST), the total community in phreatic and confined water after extreme rainfall was more regulated by stochasticity (mean NSTs = 0.946; Figure S8). Furthermore, the neutral interpretation of rare subcommunities was much higher than that of abundant subcommunities (Figure 10). Particularly, deterministic processes contributed a larger proportion to the assembly of abundant subcommunities in phreatic water after rainfall, while stochastic processes such as dispersal and drift influenced the rare taxa rather than the deterministic processes (R^2^ = 0.811). Stochastic processes had more influence on both rare (R^2^ = 0.705) and abundant taxa (R^2^ = 0.598) in confined water after extreme rainfall.

### 3.5. Microbial Potentials for Carbon, Nitrogen, Sulfur, and DMSP Cycling in Different Aquifers

**Figure 11:**
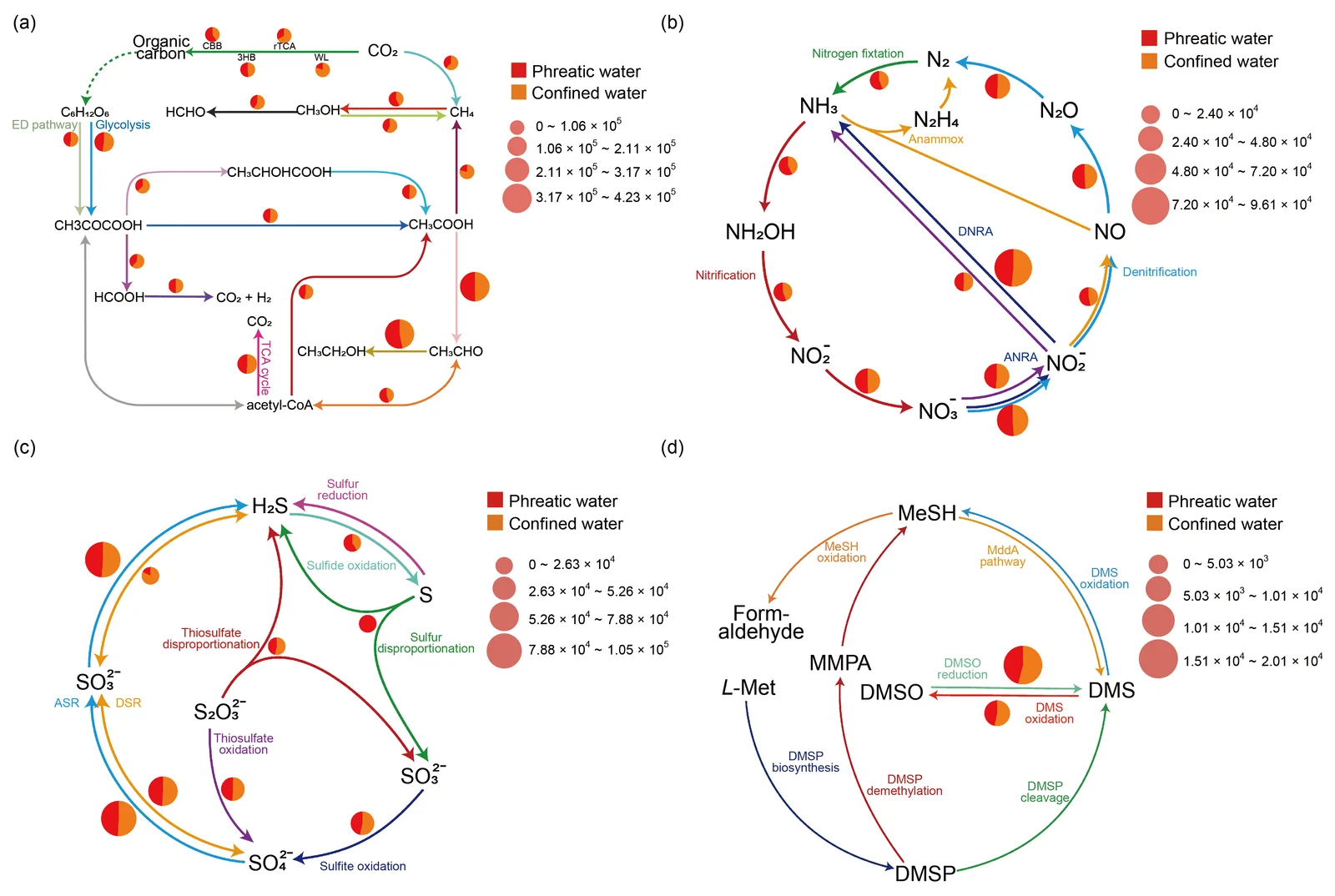
Relative abundances of the pathways involved in the carbon (**a**), nitrogen (**b**), sulfur (**c**). and DMSP (**d**) cycling in different aquifers. Red segments represent phreatic water, and orange segments represent confined water. The size of the circles indicates the total relative abundance of each pathway, with corresponding value ranges shown in the legend. (**a**) CBB, Calvin–Benson–Bassham cycle; rTCA, reductive citric acid cycle; WL, Wood–Ljungdahl pathway; 3HB, 3-hydroxypropionate bicycle. (**b**) ANRA, assimilatory nitrate reduction to ammonia; DNRA, dissimilatory nitrate reduction to ammonia; Anammox, anaerobic ammonia oxidation. (**c**) ASR, assimilatory sulfate reduction; DSR, dissimilatory sulfate reduction. (**d**) DMSP, dimethylsulfoniopropionate; MMPA, methylmecaptopropionate; MeSH, methanethiol; DMSO, dimethyl sulfoxide; *L*-Met, *L*-methionine.

Four carbon fixation pathways were identified within microbial communities. The Wood–Ljungdahl pathway (*cooS*, *fdhA*), which is capable of fixing CO_2_ into Acetyl-CoA under anaerobic conditions, as well as the reductive citric acid cycle (rTCA) that produces carbon compounds from CO_2_ and reduced compounds through an inversion of the Krebbs cycle, were enriched in confined water. The Calvin–Benson–Bassham (CBB) cycle is the main source of organic matter of autotrophic microbes. Its marker genes, *rbcL* and *rbcS*, were abundant in phreatic water (Figure 11a and Figure S9). Tricarboxylic acid cycle (TCA) and glycolysis genes did not greatly vary with aquifers, suggesting the widespread occurrence of chemoheterotrophs. The marker genes for methanogenesis, *mcrAB*, were enriched in confined water (Figure S9). These results suggested that confined water might be favorable to the production of methane.

The genes for molybdenum-dependent nitrogen fixation (*nifDHK*) were enriched in phreatic water (Figure 11b and Figure S10). Similarly, the genes for dissimilatory nitrate reduction, *napAB* and *narGHI* (nitrate→nitrite) were enriched in phreatic water, while *nirBD* and *nrfAH* (nitrite→ammonia) were enriched in confined water (Figure S10). The genes for nitrification including *amoABC* (ammonia→hydroxylamine) and hao (hydroxylamine→nitrite) were enriched in phreatic water and caused the higher nitrite accumulation. Assimilatory nitrate reduction genes (*nirA*, *narB*) were higher in the confined water compared to the phreatic water. *nirK* and *nirS* (nitrite→nitric oxide) and *norBC* (nitric oxide→nitrous oxide), involved in denitrification, were abundant genes in phreatic water.

Moreover, the SOX operon encodes a cluster of genes used by chemolithoautotrophic bacteria to oxidize reduced sulfur compounds as a source of energy. Several genes involved in this operon showed differences in phreatic and confined water (Figure 11c and Figure S11). *SoxABCXYZ* was prevalent in phreatic water. Dissimilatory sulfate reduction (DSR) was a common process in the anoxic environment. DSR is a process through which anaerobic organisms use SO_4_^2−^ as the end acceptor of electrons instead of O_2_. Notably, as markers of this process, *dsrA*, *dsrB*, *aprA*, and *aprB* were identified within groundwater bacterial communities. Both the *aprA*/*B* and *dsrA*/*B* complexes were significantly enriched in confined water. Assimilatory sulfate reduction (ASR), involves the reduction of sulfate not as an end electron acceptor but as a means of incorporating it into organic compounds like cysteine and homocysteine as the final products. Genes related to ASR such as *cysD*, *cysI*, and *cysNC* were uniformly enriched in phreatic water. The exception was *cysN*, which catalyzes the transformation of sulfate to PAPS (3′-phosphoadenosine-5′-phosphosulfate) and was abundant in confined water. Likewise, *phsABC* and *dmsABC*, encoding thiosulfate reductase and dimethyl sulfoxide reductase, were enriched in confined water. The potential for reduction of DMSO (*dmsABC*, dimethyl sulfoxide reductase) was enriched in confined water (Figure 11d and Figure S11).

## 4. Discussion

Rainfall events are associated with higher levels of TDS and NH_4_^+^-N. These increases are likely ascribed to the erosion and surface runoff instigated by rainfall, which facilitates the translocation of dissolved substances and particulate matter from the soil into the riverine and groundwater system. HCO_3_-Ca dominance indicated that water–rock interactions were the prevalent hydrogeochemical processes governing the groundwater chemical composition [62]. Geographic factors (i.e., longitude, latitude, and well depth) were responsible for the evolution of groundwater communities before rainfall, yet rainfall-induced changes in physicochemical parameters, rather than geographic factors significantly influenced abundant and rare subcommunities [63]. Abundant taxa exhibited significant responses to carbon nutrients in shallow groundwater, whereas rare taxa were governed by mineral ions in deep confined aquifers, revealing clear niche differentiation of the two subcommunities. In addition, our results revealed that abundant taxa in groundwater were environmentally less constrained and showed wider environmental thresholds in response to hydrochemical factors compared with rare taxa.

Extreme rainfall functions as a strong hydrological-pulse disturbance that breaks the long-term subsurface equilibrium of phreatic and confined aquifers across the western North China Plain. Stratification of aquifer yields divergent microbial response responses, which are governed by hydrologic connectivity, environmental filtering, and ecological assembly processes [64]. Consistent with the adaptive-cycle framework applied for interpreting aquatic microbial recovery from hydrological disturbance [65], this study verifies that alluvial groundwater microbiomes experience directional trophic succession after rain infiltration, transitioning from slow-growing oligotrophic K-strategists to r-strategists (copiotrophic or opportunistic species) with fast growth rates [24,66,67,68]. The widespread decline of bacterial alpha-diversity was observed in both phreatic water and confined water following extreme rainfall events, implying that heterotrophic microbes driven by terrestrial organic inputs could suppress native taxa [69]. The enrichment of *Gammaproteobacteria* and pathogenic genera (e.g., *Acinetobacter*, *Legionella*) in phreatic water poses critical threats to groundwater security across the densely populated North China Plain after extreme rainfall, since atmospheric precipitation promotes the infiltration of pathogens, enabling pathogens to escape soil retention and migrate into aquifers [70]. Redox-tolerant bacterial genera such as *Desulfovibrio* maintained a higher relative abundance in confined water than in phreatic water [6].

Distinguishing abundant and rare subcommunities unraveled their functional responses to extreme rainfall, further expanding existing knowledge of the subsurface microbial biosphere [5]. Rare taxa in groundwater were defined as a crucial microbial seed bank that sustained microbial diversity and community stability [71], yet this study revealed that certain rare genera were more environmentally constrained and more likely to be diluted or eliminated under rainfall disturbances. Extreme rainfall imposed severe resource reallocation, which increased the diversity of the abundant subcommunity and might further enhance the adaptation of abundant taxa against environmental fluctuations [4,72,73,74]. The changes in biomarkers verified the functional turnover of bacterial communities. Analysis revealed that abundant taxa such as oligotrophic *Vicinamibacterales* were displaced by heterotrophic *Comamonadaceae*. This was attributed to inputs of nutrients such as nitrogen and phosphorus during rainfall, which promoted the proliferation of heterotrophic groups [75]. Meanwhile, rare taxa shifted toward opportunistic *Chlamydiae* and *Massilia* [76].

Bacterial co-occurrence based on cooperative interrelation was vital to community stability even in oligotrophic environments [75,77]. The positive correlations of mutualism, parasitism, or commensalism were prevalent among abundant taxa after rainfall, whereas the negative correlations of competition for space and resources occurred more among rare taxa. More competition likely improves the stability of the network in terms of interference [78]. The identified keystone taxa in groundwater, predominantly affiliated with Proteobacteria, Firmicutes, and Actinobacteria, were mainly module hubs, which were regarded as integral elements within distinct modules and may mediate the stability of subsurface ecosystems [79,80]. Rare taxa with high closeness centrality served as ecological connectors linking distinct functional modules before rainfall. Such characteristics were more pronounced in phreatic water, and the interactions among bacterial communities in phreatic water were more susceptible to rainfall disturbances than those in confined water. Notably, compared with abundant taxa, rare taxa in groundwater exhibited stronger environmental adaptability and might contribute to community recovery under disturbance and provide a buffer against environmental fluctuations [81].

Deterministic and stochastic processes are important components of microbial community dynamics [82,83]. Increased environmental uncertainty, such as through rainfall-induced changes in soil erosion and hydrological transport, strengthens the contribution of stochastic processes to ecological community assembly [84]. We found that stochastic processes had a significant impact on shaping the rare taxa after rainfall. Enhancement of stochastic processes led to the diversification of community functions after rainfall [85,86]. Whereas deterministic processes, such as environmental filtering and species interactions, play a key role in the assembly and functioning of abundant taxa after rainfall, especially in phreatic water. The dominance of deterministic processes could cause the specialization of community functions [86].

Differentiation of carbon, nitrogen, and sulfur metabolic functional pathways reflected redox transition and material exchange shaped by subsurface aquifers [87]. Long-term anaerobic conditions facilitated carbon fixation via the WL and rTCA pathways in confined water. In the groundwater system, an increase in carbon fixation could promote methanogenesis [88]. Methanogenesis in groundwater is a significant contributor to methane emissions, especially in confined water [89]. In contrast, the CBB pathway along with nitrification and denitrification was enriched in phreatic water [90]. Moreover, the oxidation of reduced sulfur compounds was prevalent in phreatic water, while dissimilatory sulfate reduction prevailed in confined water. Such differentiation of sulfur cycling along redox gradients might be coupled with rainfall recharge dynamics [91].

Given this linkage between bacterial communities and rainfall, beginning on 29 July 2023, the NCP experienced extreme heavy precipitation due to the influence of Typhoon Doksuri. This event provides the necessary conditions for exploring the response of microorganisms under extreme rainfall. Subsequent research will reduce the randomness by extending the observation period. Meanwhile, PICRUSt provided predicted functional information derived from 16S rRNA data, which cannot reflect real-time metabolic activity and is limited by available genomes. Thus, multi-omics should be adopted in the following research to verify these functions.

## 5. Conclusions

Our study provided an investigation of the responses of bacterial communities in groundwater of the western North China Plain driven by extreme rainfall. Extreme rainfall elevated TDS, NH_4_^+^-N, and COD_Mn_, and the primary hydrochemical types gradually evolved to HCO_3_-Ca. Extreme rainfall influenced the distribution of dominant genera in groundwater and enriched tolerant bacterial communities, further causing a decline in groundwater microbial diversity. Bacterial communities in phreatic aquifers are more susceptible to disturbances induced by rainfall due to stronger connectivity with the surface. Abundant subcommunities displayed strong microbial collaborations, while rare subcommunities exhibited higher sensitivity and evolved toward opportunistic groups after extreme rainfall. Rainfall influenced the composition of rare taxa, while enhancing the influence of stochastic processes to the assembly of subcommunities. Functionally, confined water favored genes linked to anaerobic carbon fixation, methanogenesis and dissimilatory sulfate reduction, whereas genes involved in the CBB pathway, nitrification, and denitrification were enriched in phreatic water, potentially forming a hotspot for N_2_O emissions. Additionally, abundant taxa exhibited significant responses to carbon nutrients in shallow groundwater, whereas rare taxa were governed by mineral ions in deep confined aquifers after rainfall. This study improves our understanding of the dynamics of groundwater microbial communities and functions driven by extreme rainfall events and contributes to the microbial ecological prediction of ecosystem health in the western North China Plain.

## Data Availability

Complete datasets supporting the findings of this article are available from the NCBI BioProject database under accession code PRJNA1511538. Raw data are accessible by the NCBI reviewer link (https://dataview.ncbi.nlm.nih.gov/object/PRJNA1511538?reviewer=p0v8c8j146ukrptaa3vb4uhf8k (accessed on 12 August 2026)).

## Supplementary Materials

The following supporting information can be downloaded at https://www.mdpi.com/article/10.3390/microorganisms14092095/s1, Figure S1. Flowchart of experimental preparation and data analysis in this study; Figure S2. The rarefaction curves of all groundwater samples; Figure S3. Compositional difference of bacterial communities in phreatic and confined water before and after extreme rainfall. (a) Non-metric multidimensional scaling (NMDS) diagrams. (b) ANOSIM analyses; Figure S4. Diversity of abundant and rare subcommunities in phreatic and confined water before and after extreme rainfall. (a) Richness. (b) Chao1. (c) ACE. Boxplots are represented with center line: median; box limits: upper and lower quartiles; whiskers: 1.5×interquartile range; Figure S5. The heatmap shows Spearman’s correlations between the abundances of abundant/rare subcommunities and environmental variables. Mantel tests were used to evaluate the significance of Spearman’s correlations. The asterisk (*) represents *p*-values (*, *p* < 0.05; **, *p* < 0.01; ***, *p* < 0.001); Figure S6. The node-level topological features of abundant and rare taxa include (a) weighted degree and (b) closeness centrality. The asterisk (*) represents *p*-values (*, *p* < 0.05; **, *p* < 0.01; ***, *p* < 0.001); Figure S7. Neutral models evaluating the role of neutral processes in the community assembly of bacteria in phreatic and confined water before and after extreme rainfall. Blue dashed lines represent 95% confidence intervals of the neutral model prediction, R^2^ is the model goodness of fit. Green and red dots indicate abundant and rare taxa that occur more (‘above’) and less (‘below’) frequently than given by the neutral model (blue dots, ‘neutral’); Figure S8. Mean values of normalized stochasticity ratios (NST) of bacterial communities. 0.5 taken as the boundary point between more deterministic (<0.5) and more stochastic (>0.5) assemblies; Figure S9. Differences in the abundance of functional genes involved in carbon cycling. The values of differences were calculated and t-test was used to calculate the significance between two aquifers. The asterisk (*) represents *p*-values (*, *p* < 0.05; **, *p* < 0.01; ***, *p* < 0.001). The data in the phreatic water were colored in red and those for the confined water were colored in yellow; Figure S10. Differences in the abundance of functional genes involved in nitrogen cycling. The values of differences were calculated and t-test was used to calculate the significance between two aquifers. The asterisk (*) represents *p*-values (*, *p* < 0.05; **, *p* < 0.01; ***, *p* < 0.001). The data in the phreatic water were colored in red and those for the confined water were colored in yellow; Figure S11. Differences in the abundance of functional genes involved in sulfur cycling. The values of differences were calculated and t-test was used to calculate the significance between two aquifers. The asterisk (*) represents *p*-values (*, *p* < 0.05; **, *p* < 0.01; ***, *p* < 0.001). The data in the phreatic water were colored in red and those for the confined water were colored in yellow; Table S1. Standard methods used for physicochemical properties analysis in this study; Table S2. Information on sampling sites and obtained datasets; Table S3. The relative abundance of bacterial communities at the phylum level; Table S4. The relative abundance of bacterial communities at the genus level; Table S5. The composition of abundant and rare subcommunities in different groups at the phylum level; Table S6. The composition of abundant and rare subcommunities in different groups at the genus level; Table S7. The topological features of interaction networks in phreatic and confined water before and after extreme rainfall.
