# Supplementary material for: Responses of Groundwater Bacterial Communities to Extreme Rainfall in the Western North China Plain Based on 16S rRNA Sequencing
Source: Microorganisms. 2026 Sep 19;14(9):2095. doi: 10.3390/microorganisms14092095 (PMC13608952; doi:10.3390/microorganisms14092095)
Supplement: Supplementary file 1 [file microorganisms-14-02095-s001.zip › Supplementary Information.pdf]

Supplementary Material for

Responses of groundwater bacterial communities to extreme rainfall in the western North China Plain based on 16S rRNA sequencing

Authors: Yuan Gao[1,2,3*], Shengpin Li[1,3], Rui An[1,3], Yiwei Zhang[1,3], Haitao Piao[1,3], Tuoya Tai[1,3], Qianying Zhu[1,3], Qi Su[1,3], Yu Fei[1,3], Chenglong Han[1,3], Wenpeng Li[1,3], Kun Liu[1,3*]

Author affiliations:

[1]China Institute of Geo-Environment Monitoring, Beijing 100081, China

[2]School of Water Resources and Environment, China University of Geosciences (Beijing), Beijing 100083, China

[3]Key Laboratory of Mine Ecological Effects and Systematic Restoration, Ministry of Natural Resources, Beijing 100081, China

Correspondence and requests for materials should be addressed to Yuan Gao. (e-mail: gyy186349@163.com)

Conflict of interest: The authors declare no conflict of interest.

Supplemental Methods

1. Determination of Bicarbonate Ions

The concentrations of carbonate (CO_3^{2-}), bicarbonate (HCO_3^-), and hydroxide (OH^-) were determined via double-indicator titration according to DZ/T 0064.49-2021. 50.0 mL aliquot of the water sample was titrated with a standardized HCl solution (c(HCl) ≈ 0.05 mol/L) using phenolphthalein (10 g/L) and methyl orange (0.5 g/L) as indicators. The volume of acid consumed to reach the phenolphthalein endpoint (disappearance of red color) was recorded as V_1 (mL), followed by the volume consumed to reach the methyl orange endpoint (color change from yellow to orange), recorded as V_2 (mL). The mass concentrations (ρ, mg/L) were calculated as follows:

1. When $V_1 > V_2$: Hydroxide and carbonate ions are present.

$\rho(OH^-) = (c \times (V_1 - V_2) \times 17.01 \times 1000) / V$

$\rho(CO_3^{2-}) = (c \times 2V_2 \times 30.01 \times 1000) / V$

2. When $V_1 = V_2$: Only carbonate ions are present

$\rho(CO_3^{2-}) = (c \times 2V_1 \times 30.01 \times 1000) / V$

3. When $V_1 < V_2$: Carbonate and bicarbonate ions are present.

$\rho(CO_3^{2-}) = (c \times 2V_1 \times 30.01 \times 1000) /$

$\rho(HCO_3^-) = (c \times (V_2 - V_1) \times 61.02 \times 1000) / V$

4. When $V_1 = 0$: Only bicarbonate ions are present.

$\rho(HCO_3^-) = (c \times V_2 \times 61.02 \times 1000) / V$

5. When $V_2 = 0$: Only hydroxide ions are present

$\rho(OH^-) = (c \times V_1 \times 17.01 \times 1000) / V$

Where c is the concentration of the HCl standard solution (mol/L), and V is the sample volume (mL).

2. Determination of pH

pH of groundwater samples was measured by the glass-electrode method according to DZ/T 0064.5-2021. Three certified standard buffer solutions were applied for instrument calibration: potassium hydrogen phthalate (pH 4.00 at 20°C), mixed phosphate (pH 6.88 at 20°C), and sodium tetraborate (pH 9.22 at 20°C).

A precision pH meter (measurement range: 0–14; readout precision $\leqslant$ 0.02 pH units) equipped with a pH composite glass electrode and a thermometer (0–50°C, precision ± 0.1°C) was employed. Prior to measurement, the glass electrode was activated by soaking in secondary-grade laboratory water for at least 24 h. The pH meter was warmed up for 30 min before calibration, including zero adjustment and temperature compensation according to the manufacturer’s instructions. Two-point calibration was performed using standard buffer solutions: potassium hydrogen phthalate buffer was used for primary calibration for samples with pH < 7.0, followed by re-calibration with mixed-phosphate or sodium-tetraborate buffer; for samples with pH > 7.0, sodium-tetraborate buffer was used for primary calibration, and re-calibration was conducted with potassium-hydrogen-phthalate or mixed-phosphate buffer. The electrode slope was checked and maintained within 90%–100% to guarantee valid electrode performance.

After calibration, the electrode was rinsed several times with secondary-grade water and then rinsed 6–8 times with the groundwater sample. The electrode was immersed into the

sample solution, and the pH reading was recorded once the signal became stable. Field measurement was preferred for groundwater pH to minimize artefacts induced by variations of temperature and partial pressure of carbon dioxide.

3. Determination of Total Dissolved Solids

The concentration of total dissolved solids (TDS) in the groundwater samples was determined using the gravimetric method according to DZ/T 0064.9-2021. 100 mL aliquot of the water sample, filtered through a 0.45 μm membrane, was evaporated to dryness in a pre-weighed porcelain dish. The residue was then dried at either 105° C or 180° C until a constant mass was achieved (mass difference ≤ 0.2 mg). The 180° C drying temperature was employed for samples with permanent hardness, with 0.2 – 0.3 g of Na_2CO_3 added to reduce hygroscopicity. The TDS concentration (mg/L) was calculated using the formula: $\rho(TDS) = (m_1 - m_2) \times 10^6 / V$, where m_1 and m_2 represent the masses of the dish with residue and the empty dish (g), respectively, and V is the sample volume (mL).

4. Determination of Total Hardness

The total hardness of the groundwater samples was determined using the Ethylenediaminetetraacetic acid disodium salt (EDTA-2Na) titrimetric method according to DZ/T 0064.15-2021, 50.0 mL aliquot of the water sample was buffered to pH 10 with an ammonia-ammonium chloride solution and titrated with a standardized EDTA-2Na solution ($c \approx 0.01$ mol/L) using Acid Chrome Blue K-Naphthol Green B as the indicator (0.2 g acid chrome blue K and 0.5 g naphthol green B dissolved in 100 mL secondary-grade water). The endpoint was identified by a distinct color change from wine-red to pure blue. To eliminate interference from ions such as Fe^{3+}, Al^{3+}, and Cu^{2+}, masking agents including

triethanolamine, hydroxylamine hydrochloride, and sodium sulfide were added when necessary. The total hardness, expressed as $CaCO_3$ (mg/L), was calculated using the formula: $\rho(CaCO_3) = c \times (V_1 - V_2) \times 100.08 \times 1000 / V$, where c is the concentration of the EDTA-2Na solution (mol/L), V_1 and V_2 are the volumes of EDTA-2Na consumed by the sample and blank, respectively (mL), and V is the sample volume (mL).

5. Determination of Total Chromium and Chromium (VI)

Total chromium (Cr) and chromium (VI) were determined by the 1,5-diphenylcarbazide spectrophotometric method according to DZ/T 0064.17-2021. 50 mL water sample was adjusted to pH ~8 with NaOH, followed by the addition of 2.50 mL of 1,5-diphenylcarbazide reagent. After a 10 min reaction, the absorbance of the resulting purple complex was measured at 540 nm. For total chromium determination, a 50.0 mL aliquot was acidified with H_2SO_4 and oxidized with $KMnO_4$ solution (30 g/L) by boiling for 5–10 min. Excess $KMnO_4$ was reduced by adding NaN_3 solution (5 g/L) dropwise until the purple color disappeared. After cooling and neutralization, the solution was analyzed following the same procedure as for Cr (VI). The chromium concentration was calculated using the formula: $\rho(Cr) = m / V$, where $\rho(Cr)$ is the mass concentration of chromium or Cr (VI) (mg/L), m is the mass of chromium determined from the calibration curve (μg), and V is the sample volume (mL).

6. Determination of Nitrite

Nitrite (NO_2^-) was determined by spectrophotometry according to DZ/T 0064.60-2021. 50.0 mL water sample was mixed with 2.0 mL of a sulfanilamide-α-naphthylamine reagent mixed reagent consisting of equal volume mixture of 0.8 g/L sulfanilamide solution (dissolved in acetic acid solution) and 0.2 g/L α naphthylamine solution (dissolved in acetic

acid solution). After a 10 min reaction, the absorbance of the resulting purple azo dye was measured at 540 nm against a reagent blank. The nitrite concentration was calculated using the formula: $\rho(NO_2^-) = m / V$, where $\rho(NO_2^-)$ is the mass concentration of nitrite (mg/L), m is the mass of nitrite determined from the calibration curve (μg), and V is the sample volume (mL).

7. Determination of Oxygen Consumption

Oxygen Consumption (COD_{Mn}) was determined by the acidic potassium permanganate titrimetric method according to DZ/T 0064.68-2021. 100 mL water sample was acidified with 5 mL of sulfuric acid solution and treated with 10.0 mL of standardized potassium permanganate solution ($c \approx 0.01$ mol/L). The mixture was heated in a boiling water bath for 30 min to oxidize the organic matter. Subsequently, 10.0 mL of sodium oxalate standard solution ($c = 0.0100$ mol/L) was added to reduce the excess permanganate. The remaining oxalate was back-titrated with the potassium permanganate solution to a faint pink endpoint. The COD_{Mn} concentration was calculated using the formula: $\rho(O_2) = c \times (V_1 - V_2) \times 8 \times 1000 / V$, where $\rho(O_2)$ is the mass concentration of oxygen (mg/L), c is the concentration of the potassium permanganate solution (mol/L), V_1 is the total volume of potassium permanganate solution consumed (mL), V_2 is the volume of potassium permanganate solution equivalent to the added sodium oxalate (mL), and V is the sample volume (mL).

8. Determination of Fluoride, Chloride, Nitrate and Sulfate

Fluoride (F^-), chloride (Cl^-), nitrate (NO_3^-) and sulfate (SO_4^{2-}) in groundwater were determined by ion chromatography according to HJ 84-2016. Samples were filtered through a 0.45 μm aqueous phase membrane filter prior to injection; samples containing hydrophobic organics or heavy metal interferences were cleaned with RP/(C_{18}) and H/Na

type solid phase pretreatment columns, respectively. A suppressed conductivity ion chromatograph equipped with an anion exchange separation column and corresponding guard column was adopted; the injection volume was 25 μL, and carbonate based eluent was used for isocratic elution. Qualification was performed according to analyte retention time, and quantification was based on peak area response. Mixed anion calibration standard series were prepared by serial dilution of stock standard solutions.

The mass concentration ρ (mg/L) of target anions was calculated as: $\rho = [(h - h_0 - a) / b] \times f$ Where: h = peak area of sample; h_0 = peak area of laboratory blank; a = intercept of calibration regression equation; b = slope of calibration regression equation; f = sample dilution factor.

9. Determination of Ammonia Nitrogen

Ammonia nitrogen (NH_4^+-N) was determined by Nessler's reagent spectrophotometry according to HJ 535-2009. 50.0 mL aliquot of the water sample was mixed with 1.0 mL of potassium sodium tartrate solution (ρ = 500 g/L) to mask interfering metal ions, followed by the addition of 1.5 mL of Nessler's reagent ($HgCl_2$-KI-KOH). After a 10 min reaction, the absorbance of the resulting yellow-brown complex was measured at 420 nm using a 20 mm cuvette. The ammonia nitrogen concentration was calculated using the formula: $\rho(\mathrm{N}) = (A_s - A_b - a) / (b \times V)$, where ρ(N) is the mass concentration of ammonia nitrogen (mg/L), A_s and A_b are the absorbances of the sample and blank, respectively, a and b are the intercept and slope of the calibration curve, and V is the sample volume (L).

10. Determination of Arsenic and Selenium

Arsenic (As) and selenium (Se) in groundwater were determined by atomic fluorescence

spectrometry according to HJ 694-2014. For dissolved-phase analysis, water samples were filtered through a 0.45 μm aqueous membrane filter immediately after sampling; total-element analysis used unfiltered raw water. Samples were preserved by adding hydrochloric acid, with a holding time of 14 days. For sample digestion, 50.0 mL homogenized sample was transferred into a 150 mL Erlenmeyer flask, followed by addition of 5 mL nitric-perchloric mixed acid (volume ratio 1:1). The solution was heated on an electric hot-plate until white fumes appeared. After cooling, 5 mL hydrochloric acid solution was added, and heating continued until brown-yellow fumes were completely expelled. The digest was cooled, quantitatively transferred into a 50 mL volumetric flask, and diluted to volume with deionized water. Prior to instrumental measurement, an aliquot of digested solution was treated with 2 mL hydrochloric acid solution and 2 mL thiourea-ascorbic acid mixed solution (50 g/L thiourea + 50 g/L ascorbic acid), then kept for 30 min for pre-reduction at room temperature (30°C water-bath incubation if room temperature <15°C).

An atomic fluorescence spectrometer equipped with As- and Se-specific hollow-cathode lamps was adopted. Instrument operating parameters: negative high-voltage 260–300 V; lamp current: As 40–60 mA, Se 80–100 mA; atomizer pre-heating temperature 200°C; carrier-gas (argon, ⩾99.999%) flow rate 400 mL/min; shield-gas flow rate 900–1000 mL/min; peak-area integration mode. Hydrochloric acid solution served as carrier solution, and potassium borohydride solution B (2.0 g/100 mL, dissolved in 0.5 g/100 mL sodium hydroxide solution, freshly prepared) was used as reductant. External calibration standard series were prepared from As and Se working standard solutions. The mass concentration of target elements was calculated as: $\rho = (\rho_1 \times f \times V_1) / V$, where ρ = measured element

concentration (μg/L); ρ_1 = element concentration read from calibration curve (μg/L); f = dilution factor of digested solution; V_1 = final constant-volume of tested aliquot (mL); V = volume of digested sample taken for sampling (mL).

11. Determination of Potassium, Calcium, Sodium and Magnesium

Potassium (K), calcium (Ca), sodium (Na) and magnesium (Mg) in groundwater were determined by inductively coupled plasma optical emission spectrometry (ICP OES) according to HJ 776-2015. For dissolved fraction analysis, water samples were immediately filtered through a 0.45 μm aqueous phase membrane filter, and filtrates were acidified with nitric acid to achieve 1% (v/v) nitric acid matrix. For total element analysis, unfiltered raw samples were acidified with nitric acid and digested on an electric hot plate; 5 mL nitric acid was added per 100 mL sample, heated gently to near dryness without boiling, and redissolved with dilute nitric acid after cooling. Multi element mixed calibration standards were prepared by serial dilution of 1000 mg/L single element stock solutions for K, Ca, Na and Mg, with 1% (v/v) nitric acid as the matrix matching diluent. The ICP OES instrument was operated under recommended conditions: radio frequency power 1150 W, nebulizer gas flow 0.7 L/min, auxiliary gas flow 1.0 L/min, coolant gas flow 12.0 L/min; characteristic emission spectral lines were selected for each target element, and spectral overlap interferences were corrected using interference coefficient method when necessary. Quantification was performed based on emission intensity. The element mass concentration ρ (mg/L) was calculated as: $\rho = (\rho_1 - \rho_2) \times f$, where ρ_1 = measured concentration from calibration curve (mg/L), ρ_2 = corresponding blank concentration (mg/L), f = sample dilution factor.

12. Determination of Iron, Manganese, Copper, Zinc, and Aluminium

Iron (Fe), manganese (Mn), copper (Cu), zinc (Zn), aluminium (Al) in groundwater were determined by inductively coupled plasma-mass spectrometry (ICP-MS) according to HJ 700-2014. For dissolved-element measurement, water samples were immediately filtered through a 0.45 μm aqueous-phase membrane filter after collection, and filtrates were acidified with concentrated nitric acid to $pH < 2$. For total-element analysis, unfiltered raw water samples were digested by electric hot-plate digestion: 100 mL homogenized sample was transferred into a 250 mL PTFE beaker, 2 mL nitric acid and 1.0 mL hydrochloric acid were added, digested at temperature ≤85℃ until sample volume was reduced to approximately 20 mL, cooled, transferred and diluted to 50 mL with ultrapure water. Ultrapure water and high-purity grade nitric/hydrochloric acid were adopted for reagent preparation. Mixed multi-element calibration standards were serially diluted from 1000 mg/L single-element stock solutions using 2% (v/v) nitric acid matrix-matched diluent. Selecting the internal standard element, and internal-standard solution was added online via peristaltic pump during measurement. ICP-MS instrument was tuned before analysis; polyatomic-ion spectral interferences were eliminated by collision-reaction-cell technology or interference-correction equations. The mass concentration of target elements was calculated as: $\rho = (\rho_1 - \rho_2) \times f$, where ρ = final sample mass concentration, ρ_1 = concentration read from calibration curve, ρ_2 = laboratory blank concentration, f = sample dilution factor.

Supplementary Figures

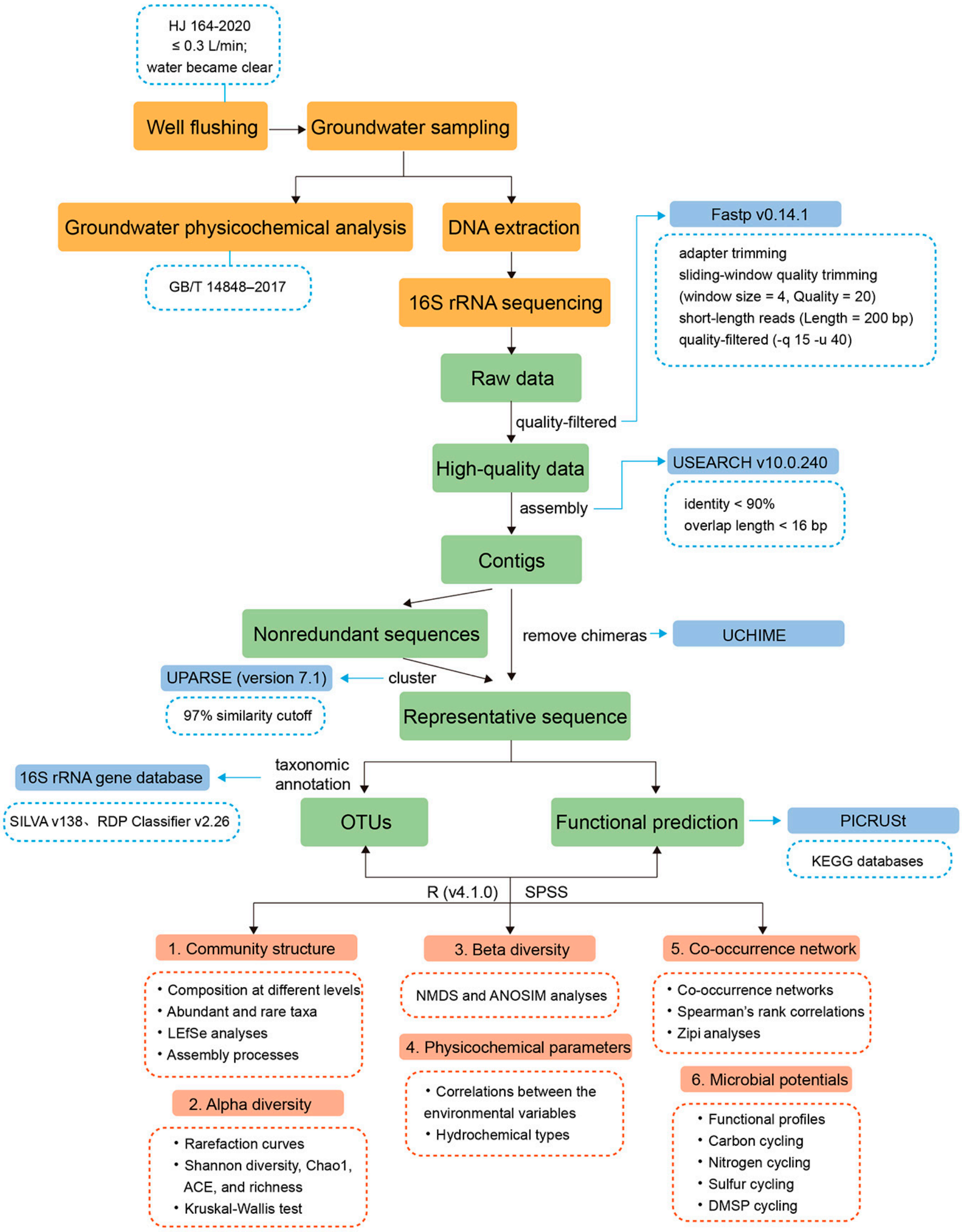

Figure S1. Flowchart of experimental preparation and data analysis in this study.

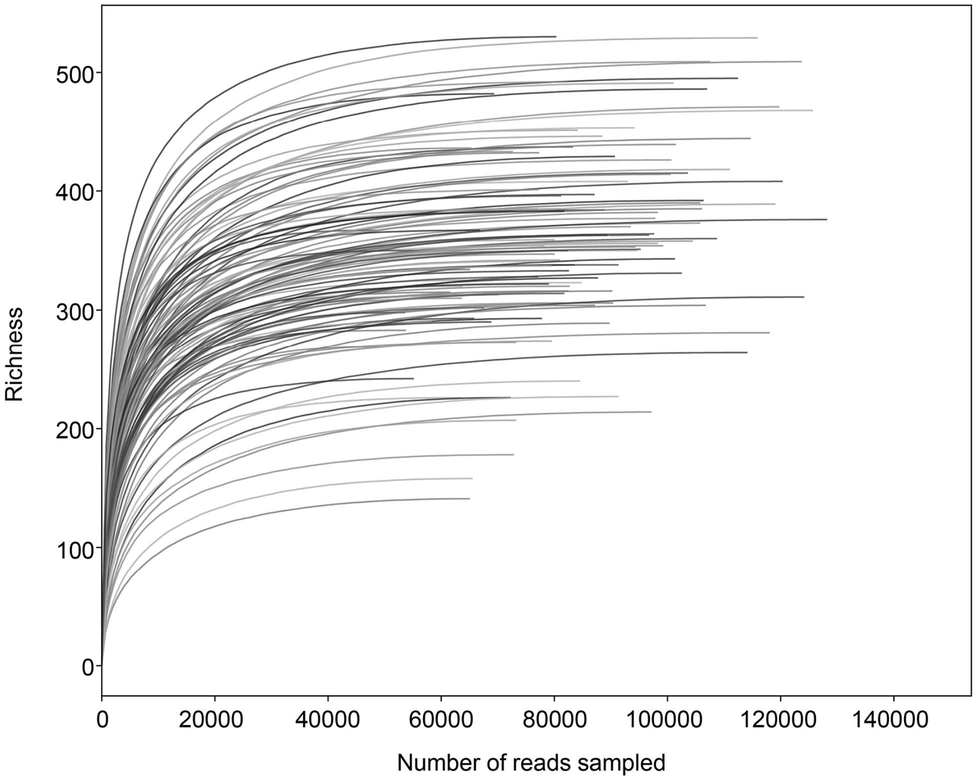

Figure S2. The rarefaction curves of all groundwater samples.

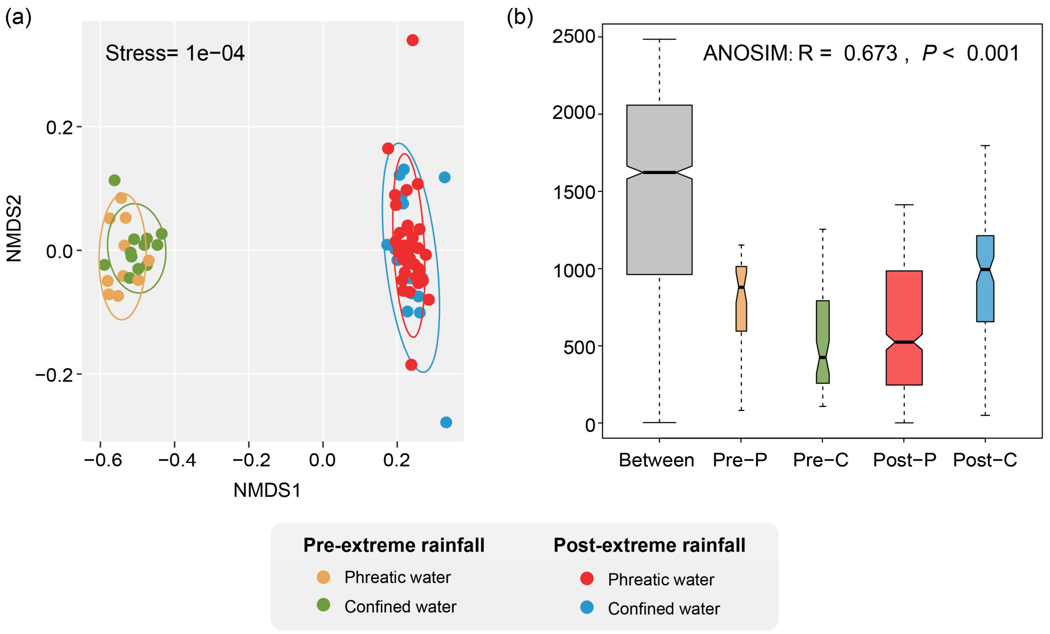

Figure S3. Compositional difference of bacterial communities in phreatic and confined water before and after extreme rainfall. (**a**) Non-metric multidimensional scaling (NMDS) diagrams. (**b**) ANOSIM analyses.

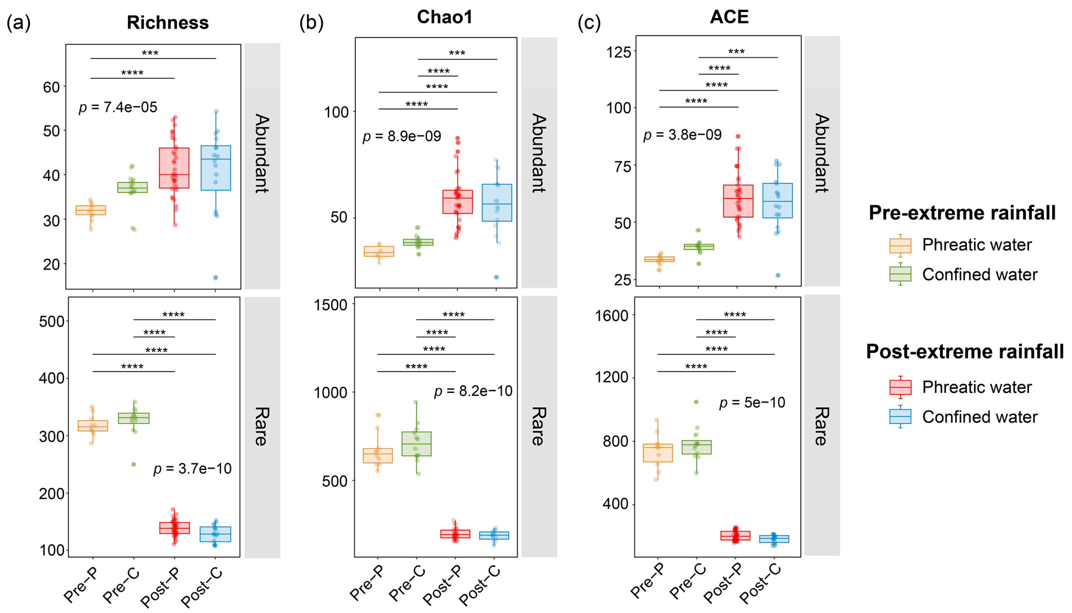

Figure S4. Diversity of abundant and rare subcommunities in phreatic and confined water before and after extreme rainfall. (**a**) Richness. (**b**) Chao1. (**c**) ACE. Boxplots are represented with center line: median; box limits: upper and lower quartiles; whiskers: 1.5×interquartile range.

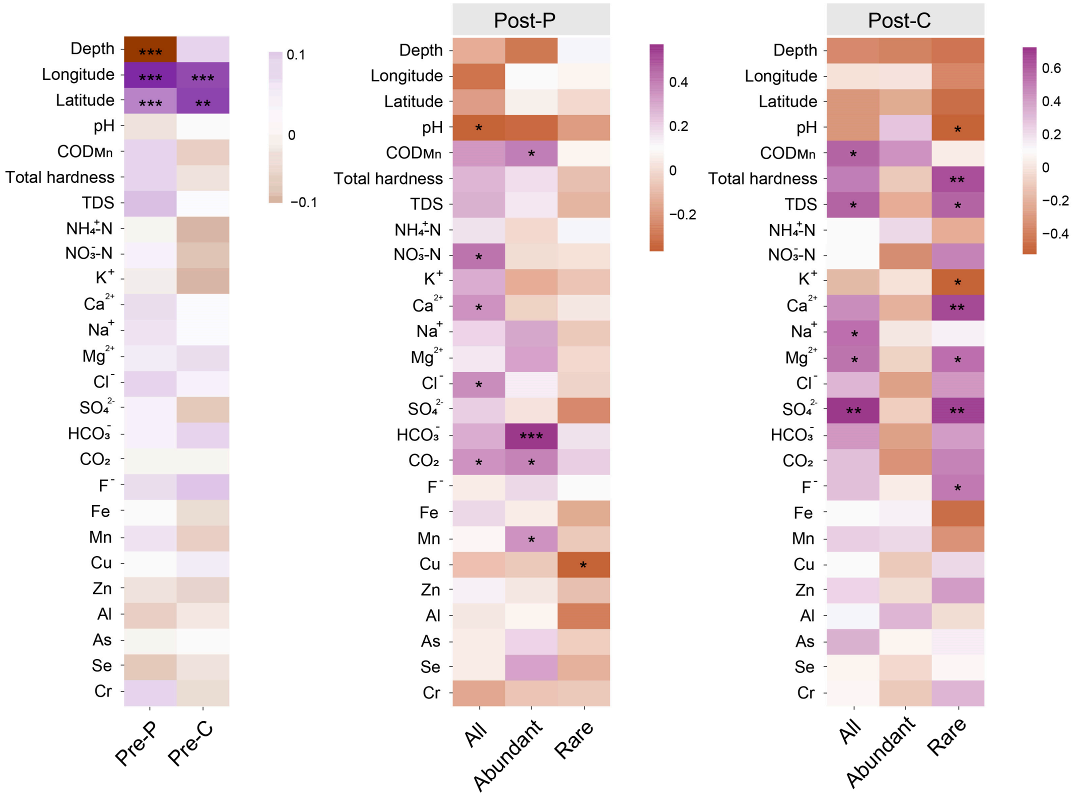

Figure S5. The heatmap shows Spearman's correlations between the abundances of abundant/rare subcommunities and environmental variables. Mantel tests were used to evaluate the significance of Spearman's correlations. The asterisk (*) represents *p*-values (*, $p < 0.05$; **, $p < 0.01$; ***, $p < 0.001$).

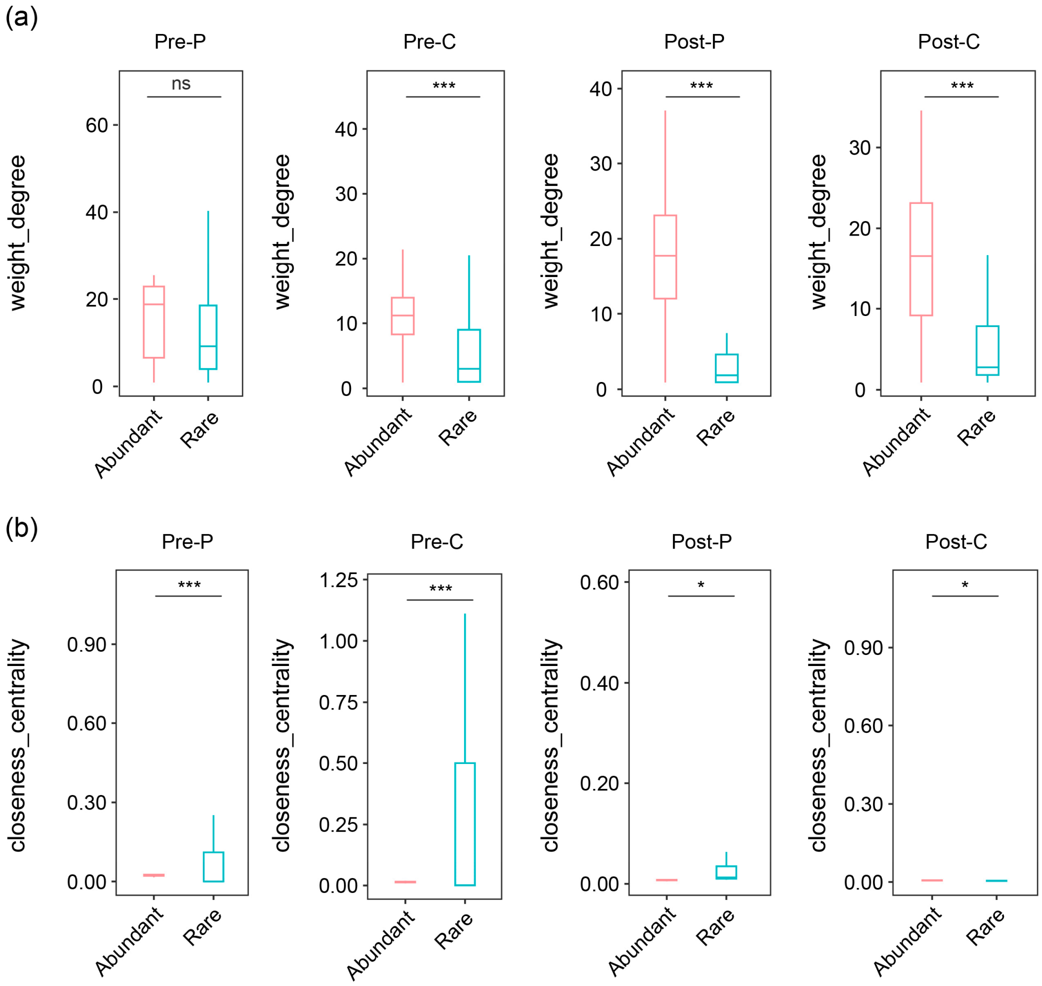

Figure S6. The node-level topological features of abundant and rare taxa include (**a**) weighted degree and (**b**) closeness centrality. The asterisk (*) represents p-values (*, $p < 0.05$; **, $p < 0.01$; ***, $p < 0.001$).

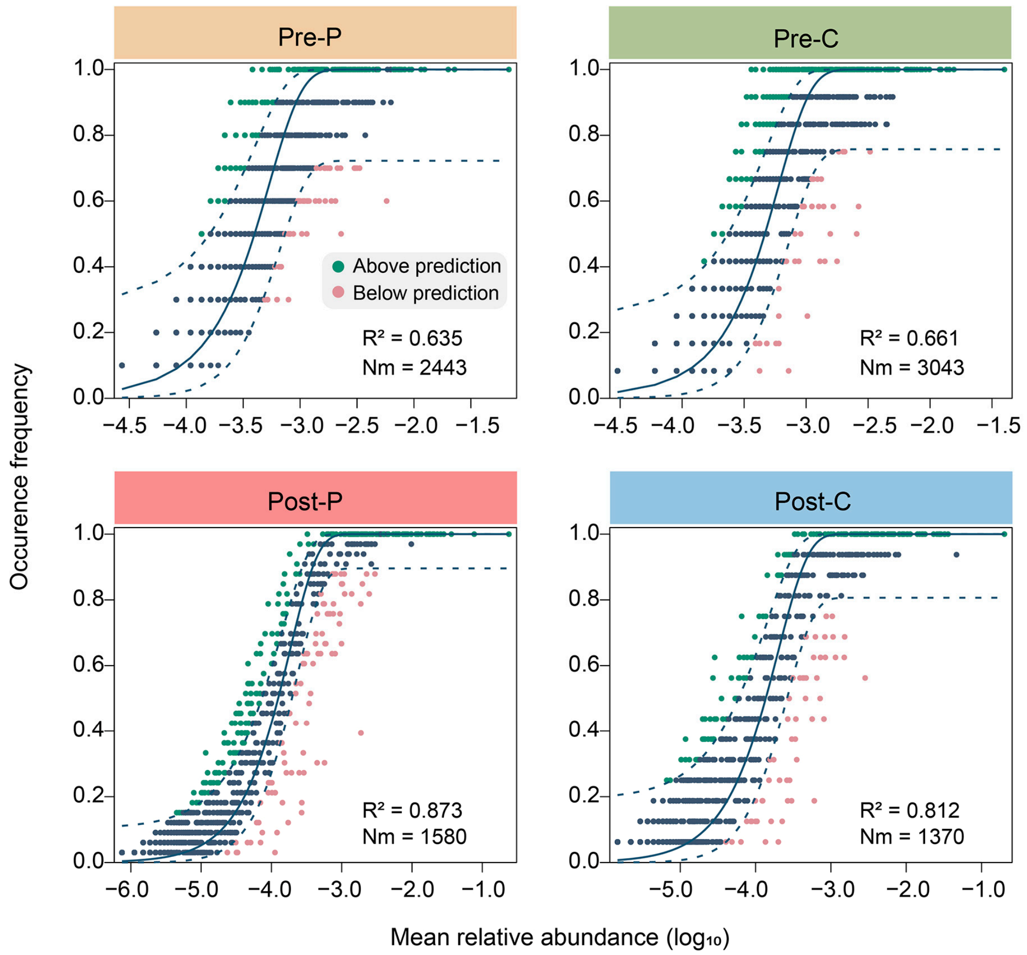

Figure S7. Neutral models evaluating the role of neutral processes in the community assembly of bacteria in phreatic and confined water before and after extreme rainfall. Blue dashed lines represent 95% confidence intervals of the neutral model prediction, R^2 is the model goodness of fit. Green and red dots indicate abundant and rare taxa that occur more ('above') and less ('below') frequently than given by the neutral model (blue dots, 'neutral').

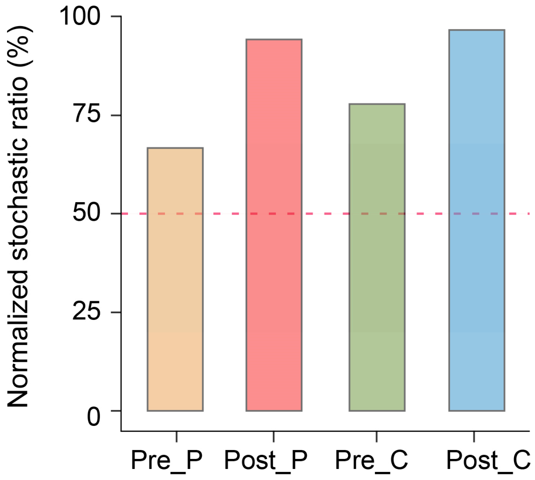

Figure S8. Mean values of normalized stochasticity ratios (NST) of bacterial communities. 0.5 taken as the boundary point between more deterministic (<0.5) and more stochastic (>0.5) assemblies.

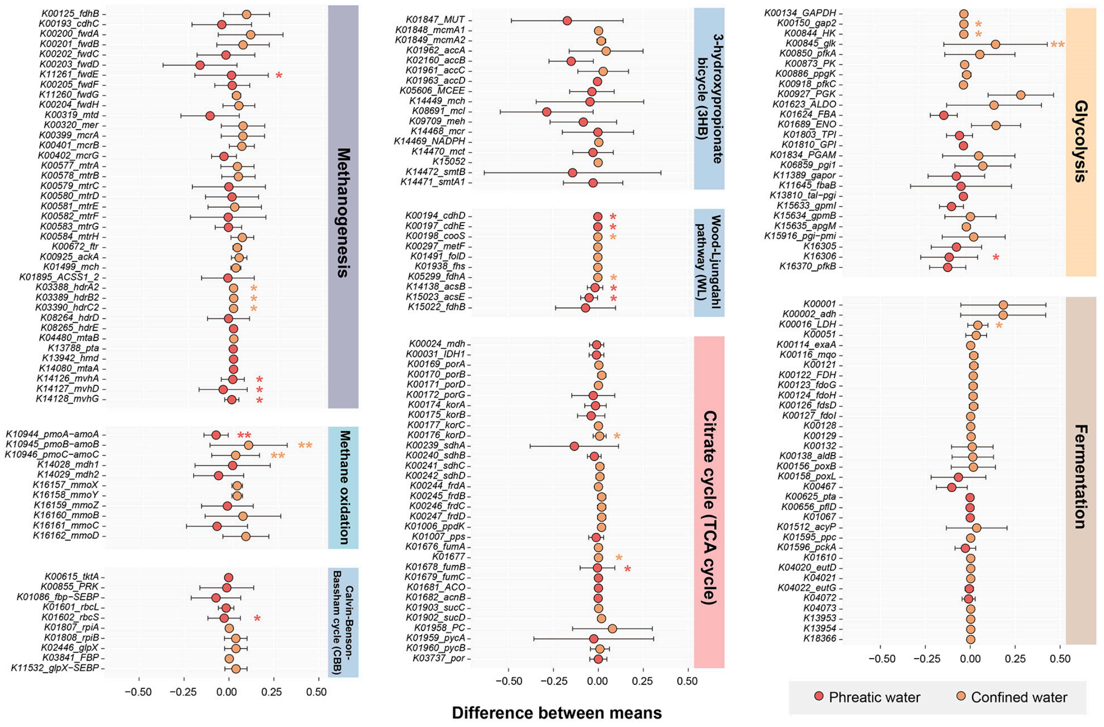

Figure S9. Differences in the abundance of functional genes involved in carbon cycling. The values of differences were calculated and t-test was used to calculate the significance between two aquifers. The asterisk (*) represents *p*-values (*, $p < 0.05$; **, $p < 0.01$; ***, $p < 0.001$). The data in the phreatic water were colored in red and those for the confined water were colored in yellow.

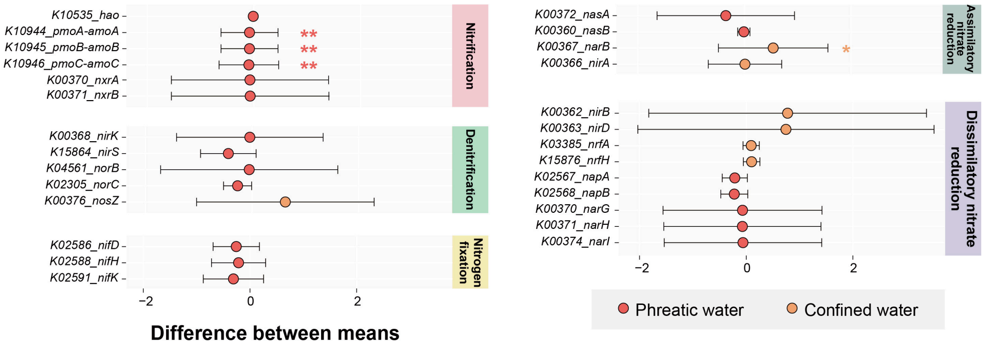

Figure S10. Differences in the abundance of functional genes involved in nitrogen cycling. The values of differences were calculated and t-test was used to calculate the significance between two aquifers. The asterisk (*) represents p-values (*, $p < 0.05$; **, $p < 0.01$; ***, $p < 0.001$). The data in the phreatic water were colored in red and those for the confined water were colored in yellow.

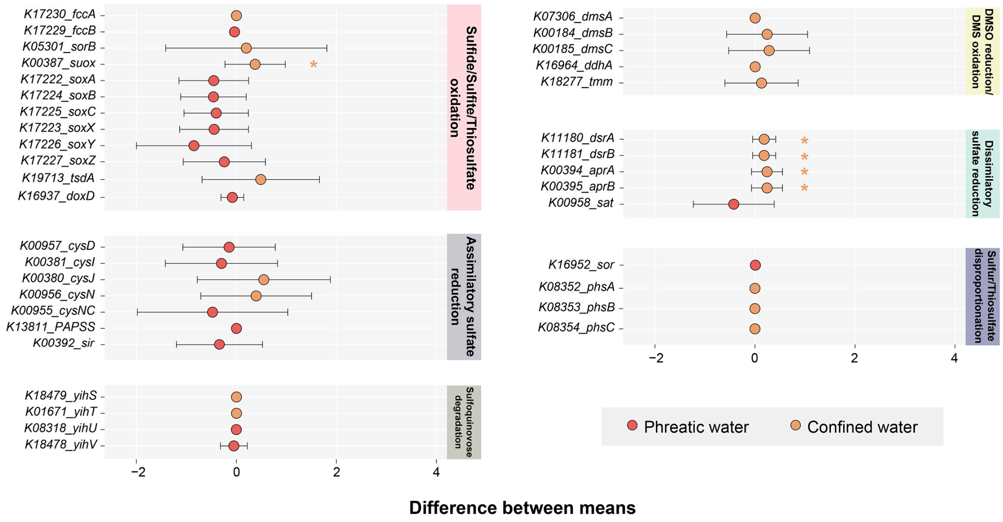

Figure S11. Differences in the abundance of functional genes involved in sulfur cycling. The values of differences were calculated and t-test was used to calculate the significance between two aquifers. The asterisk (*) represents *p*-values (*, $p < 0.05$; **, $p < 0.01$; ***, $p < 0.001$). The data in the phreatic water were colored in red and those for the confined water were colored in yellow.

Supplementary Tables

Table S1. Standard methods used for physicochemical properties analysis in this study.

Table S2. Information on sampling sites and obtained datasets.

Table S3. The relative abundance of bacterial communities at the phylum level.

Table S4. The relative abundance of bacterial communities at the genus level.

Table S5. The composition of abundant and rare subcommunities in different groups at the phylum level.

Table S6. The composition of abundant and rare subcommunities in different groups at the genus level.

Table S7. The topological features of interaction networks in phreatic and confined water before and after extreme rainfall.
